# Comparative Analysis of Plasma Biomarkers of Alzheimer’s Disease and Frontotemporal Dementia: The Dual Role of Soluble Fractalkine as a Biomarker of Frontotemporal Dementia and Its Neuroprotective Effects in Cortical Neurons “In Vitro”

**DOI:** 10.3390/life16091554

**Published:** 2026-09-16

**Authors:** José Joaquín Merino, José Julio Rodríguez-Arellano, Xavier Busquets, Ana I. Flores, Adolfo Toledano

**Affiliations:** 1 Departamento de Farmacología, Farmacognosia y Botánica, Facultad de Farmacia, Universidad Complutense de Madrid (UCM), 28040 Madrid, Spain; 2 Instituto Pluridisciplinar, Universidad Complutense de Madrid (UCM), 28040 Madrid, Spain; 3 Grupo de Medicina Regenerativa, Instituto de Investigación Sanitaria Hospital 12 de Octubre (imas12), 28041 Madrid, Spain; aflores@h12o.es; 4 Functional Neuroanatomy Group, IKERBASQUE, Basque Foundation for Science, 48009 Bilbao, Spain; j.rodriguez-arellano@ikerbasque.org; 5 Department of Neurosciences, Medical Faculty, University of the Basque Country (UPV/EHU), 48009 Leioa, Spain; 6 Laboratory of Molecular Cell Biomedicine, Department of Biology, Faculty of Medicine, University of the Balearic Islands, 07122 Palma de Mallorca, Spain; xavier.busquets@uib.es; 7 Instituto Cajal, CSIC, 28002 Madrid, Spain; atoledano@cajal.csic.es

**Keywords:** CX3CR1/fractalkine, chemokines, Alzheimer’s Disease (AD), frontotemporal dementia (FTD), neuroinflammation, cortical neurons “in vitro”, chemokines, neuropathology, tau, β-amyloid, biomarkers of cognitive disfunction, hippocampal involution

## Abstract

Frontotemporal lobar degeneration (FTD) and Alzheimer’s disease (AD) are proteinopathies characterized by the abnormal accumulation and aggregation of specific proteins like Aβ-42 deposits, p-tau accumulation, and abnormal cytosolic aggregation of TAR DNA-binding protein 43 (TDP-43) in FTD patients. Recently, the identification of neuroinflammatory mediators as predictors of cognitive decline has gained attention. We have compared several plasma biomarkers of cognitive impairment between AD and FTD patients using Enzyme-Linked Immunosorbent Assay (ELISA) (pg/mL), including CX3CR1 and soluble fractalkine (sFK, also termed CX3CL1), TDP-43, neurofilament light chain M (NfL M), p-tau217, and GFAP (glial fibrillary acidic protein). Chemokines are HIV-1 co-receptors that facilitate the spread of HIV-1 infection and induce apoptosis in the brain. Whilst these chemokines promote neuronal survival and regulate neuron-glia interactions, they also contribute to neurodegeneration. Fractalkine, also known as CX3CL1, is a delta chemokine that binds to its CX3CR1 chemokine receptor. As a membrane isoform, fractalkine can be released in a soluble form by damaged neurons under either inflammatory and/or excitotoxic conditions; since neuroinflammation contributes to neurodegeneration and dementia, we compared these CX3CR1/sFK delta chemokine levels in seropositive patients (with suppressed viral loads) and without neurodegeneration to age-matched controls. This was done in order to study whether inflammation could upregulate these chemokines in the absence of cognitive impairment. To our knowledge, this is the first study showing that increased plasma levels of CX3CR1 and soluble fractalkine could be associated with FTD pathology as compared to control subjects (without neurodegeneration). However, peripheral NfL M, GFAP, and p-tau217 levels did not differ between AD and FTD patients. Post-mortem analysis of human FTD brains revealed anatomical changes, including hippocampal involution and tau deposits. Given that soluble fractalkine can contribute to cognitive impairment while also exerting protective effects against brain insults, we did an additional (independent experiment) in cortical neurons in vitro under LPS-induced neurotoxicity. We confirmed that adding recombinant fractalkine for 24 h prevented lipopolysaccharide (LPS)-induced apoptosis in cortical neurons at 7 days in vitro (DIV); moreover, twenty-four hours after LPS treatment, sFK prevented neuronal apoptosis by decreasing caspase-3 activity and exerted neuroprotective effects against inflammation. Our findings suggest that fractalkine mitigates LPS-induced neuronal injury by limiting apoptosis and inflammatory responses. Furthermore, our findings suggest for the first time that elevated circulating sFK levels may indirectly be associated with FTD pathology as compared to healthy controls. However, sFK could also reflect compensatory protective responses to neuronal injury.

## 1. Introduction

The two most common forms of late-onset dementias are AD and FTD [1]. Many basic and translational studies have examined the causes and dementia-related mechanisms, as well as therapeutic interventions, for both AD and FTD. Currently, the diagnosis relies on clinical symptoms and neuroimaging. and cerebrospinal fluid (CSF) biomarkers, all of which are costly and time-consuming. AD and FTD are characterized by the aggregation of misfolded proteins, such as amyloid-β (Aβ) plaques and hyperphosphorylated tau deposits that form neurofibrillary tangles (NFTs). Furthermore, neuroinflammation, neuronal apoptosis, and disrupted synaptic communication also contribute to dementia [2,3]. Clinical subtypes of FTD include behavioral variant, frontal variant, progressive nonfluent aphasia, semantic dementia, and logopenic primary progressive aphasia [4,5]. TDP-43 is a crucial RNA/DNA-binding protein involved in metabolism [6]. Under normal conditions, TDP-43 is found in the nucleus. However, in FTD, TDP-43 undergoes delocalization and finally aggregates into cytoplasmic inclusions [7]. The accumulation of Aβ, NFTs, and glial fibrillary acidic protein (GFAP) is characteristic of rodent models of AD and evidently appears in the brain of AD patients [8,9,10,11,12].

The combination of neuropsychological tests and biomarker determination enables the identification of the onset of cognitive decline (e.g., cortical versus subcortical dementias) [13]. For instance, neurofilament light (NfL M), a marker of axonal damage, is detected in altered neuroglial processes and can be detected in blood and CSF [10,11,12]. NfL M levels increase progressively with age, and they are affected by subclinical brain injuries and comorbidities [14]. Tau has been detected in FTD neurofibrillary lesions, corticobasal degeneration, and progressive supranuclear palsy [15,16]. Phosphorylated tau (p-tau217) reflects peri-plaque synaptic pathology at pre- and post-synaptic sites surrounding Aβ deposits [17,18]. However, this relationship is not universal. Astroglial alterations, as determined by GFAP, may first arise in AD, particularly in the entorhinal cortex [9,10,11,12]. The identification of neuroinflammatory mediators in AD or FTD has gained attention due to their potential for the detection and monitoring of dementia progression [19]. Because neuroinflammation, including chemokine signaling, contributes to cognitive decline, we investigated the potential role of the CX3CR1/CX3CL1 (also termed fractalkine, sFK) axis as a biomarker of cognitive dysfunction, particularly in patients with FTD. Fractalkine (CX3CL1) is a delta chemokine released by neurons that binds to its receptor (CX3CR1) on microglial cells [20]. This neuronal transmembrane-anchored chemokine can be cleaved by metalloproteases in response to inflammatory and excitotoxic insults, generating a soluble isoform. Fractalkine exerts neuroprotective and chemotactic functions. However, the roles of both isoforms in neurodegenerative pathophysiology remain unclear [21]. Studying new plasma biomarkers (CX3CR1/sFK and TDP-43, GFAP, NfL M) as predictors of cognitive dysfunction contributes to our understanding of the pathophysiology of dementia, which is a key step towards developing new, effective therapies [22,23].

Conversely, HIV-1 primarily enters immune cells through the CD4 receptor, which induces conformational changes that enable the virus to bind to co-receptors (such as CCR5 and/or CXCR4), fusing intimately with the host cell. Since chemokines and their receptors contribute to HIV-1 neuropathogenesis by inducing synaptic and dendritic alterations, neurodegeneration, and cognitive impairment [23], we evaluated peripheral CX3CR1/sFK levels in HIV-1 seropositive patients with suppressed viral loads without cognitive impairment. The assessment of these delta chemokines in HIV-1 seropositive patients will help to determine whether their levels are up-regulated because of chronic inflammation (in the absence of cognitive impairment), since these patients are cognitively healthy. Thus, the inclusion of HIV-1 positive patients without cognitive impairment may help to discriminate whether alterations in these delta chemokines are specifically associated with cognitive impairment in either AD or FTD patients, rather than with a chronic inflammatory state.

### Aims

The primary aim of this study is to compare several plasma biomarkers of cognitive dysfunction (CX3CR1/sFK, TDP-43, p-tau217, GFAP, and NfL M) in patients with AD and FTD with age-matched controls and HIV patients (with suppressed viral loads, <50 copies/mL) without cognitive impairment.

After finding that sFK is elevated in patients with FTD, we investigated whether the recombinant soluble fractalkine protein could protect cortical neurons against caspase-3-dependent apoptosis and increase survival in lipopolysaccharide (LPS)-induced inflammatory conditions at 7 days in vitro (DIV), given its dual neuroprotective role and its use as a biomarker of cognitive dysfunction.

## 2. Materials and Methods

### 2.1. Clinical Selection of Participants

A total of 53 patients with AD, 53 patients with FTD, and controls without cognitive impairment were diagnosed and enrolled by neurologists. The participants were Caucasian, between 54 and 77 years old; the control subjects were of the same age range, as were the participants without dementia (n = 53, ages between 54 and 75 years old). Thus, a total of 227 patients were enrolled: 159 patients with AD or FTD, 34 seropositive patients, and 34 controls without cognitive impairment. Since chemokines are HIV-1 co-receptors, we also compared delta levels of peripheral chemokines (CX3CR/soluble fractalkine) and p-tau between 34 HIV-1 seropositive individuals with suppressed viral loads (less than 50 copies/mL) and cognitively normal, age-matched controls without HIV-1 (n = 34). Table 1 shows the demographics of the cohorts and the Mini-Mental State Examination (MMSE) scores for the neuropsychological assessment of cognitive impairments. We measured several biomarkers in peripheral blood by ELISA in all groups, including the CX3CR1/soluble fractalkine chemokine axis, TDP-43, p-tau217, NfL M, and GFAP, to evaluate their possible relationship with MMSE scores.

All participants signed informed consent, and the investigation was carried out in accordance with the Declaration of Helsinki (1975, revised in 2013). According to point 23 of the Declaration, this study was conducted in accordance with the Declaration of Helsinki (1974, updated in 2000) and was approved by the Ethics Committee CEIC of HULP PI-837 on 6 May 2009 (IdiPaz, HULP: Hospital Universitario La Paz, Madrid, Spain). The study meets national and international guidelines.

#### 2.1.1. Inclusion Criteria: Clinical Selection of Patients

Patients aged 54 to 77 years old with at least 6 years of formal education, the ability to provide informed consent, and sufficient language, visual, and auditory acuity to complete cognitive tests were included in the study. All subjects underwent blood sampling within a maximum interval of one year and were evaluated using neuropsychological tests within the normative range for their age, sex, and educational background. The MMSE is a validated tool for assessing cognitive function in Spanish-speaking populations as well [24]. Higher scores on this test reflect a good cognitive status, while lower scores indicate cognitive dysfunction in patients with AD or FTD. A total of 53 patients were included in both groups. Healthy control subjects (HC) underwent the same Mini-Mental Test and achieved scores between 27 and 30 (n = 53), confirming the absence of cognitive impairment [24] (see Table 1); in addition, neuropathological, histological [25] and peripheral biomarkers by ELISA in all groups [26]. The INCDS-ADRDA criteria were followed for AD diagnoses and validated by using Mini-Mental scores and neuropsychological tools [27,28,29]. We also used the Rascovsky criteria for FTD enrollment [30]. In the Mini-Mental Test, AD and FTD patients with cognitive impairment showed scores below 27, which is representative of cognitive impairment. Additionally, we used Raman analysis of peripheral blood samples from a previously published study as an additional criterion to confirm AD diagnoses, as AD patients showed altered stages of protein beta conformation, which identify and differentiate AD from FTD blood samples and control subjects [29]. A recent systematic review confirmed that the NINCDS–ADRDA criteria were used in 56% of AD studies without stage interventions, and the MMSE was the most frequently used tool for cognitive assessment (86%). Moreover, the age range of the selected AD patients was 45 to 95 years, with 50 as the most common lower age limit [31], consistent with our selection criteria for the study cohort.

The Rascovsky criteria for diagnosing behavioral variant FTD are based on the progressive emergence and recurrence of at least three core clinical features, including cognitive decline in functional ability [30]. The FTD diagnosis includes behavioral alterations such as disinhibition, apathy, loss of sympathy/empathy, compulsive behavior, hyperorality, and cognitive alteration (dysexecutive profile with preservation of episodic memory and visuospatial ability) [30,31].

#### 2.1.2. Exclusion Criteria

Patients with psychiatric or neurological illnesses (e.g., ELA or disabling stroke) or under 54 years of age were excluded. We did not enroll those taking anticoagulants, receiving immunosuppressive therapy, or with reversible systemic disorders (e.g., thyroid disease or B12 deficiency). Finally, we excluded those with alcohol abuse, metabolic syndrome, diabetes, hypertension, or hyperlipemia.

### 2.2. ELISAs for Biochemical Markers: Blood Collection for Biomarker Analysis

Blood samples were collected from all study groups to measure biomarkers. The samples were collected in the morning and placed in EDTA tubes. The plasma samples were aliquoted and stored at −80 °C for further biochemical determination of the biomarkers by ELISA (pg/mL or ng/mL). ELISA determinations in plasma samples were quantified for the CX3CR1/soluble fractalkine axis, TDP-43, p-tau217, NfL M, and GFAP proteins in AD, FTD, and their respective control groups (without cognitive impairment), as well as in HIV-1-seropositive patients with suppressed viral load but without cognitive dysfunction and their respective controls.

#### Soluble Fractalkine

The levels of sFK were quantified using an R&D ELISA kit (R&D Systems, Minneapolis, MN, USA) for human samples. Briefly, the plate was coated overnight at 25 °C with 1 g/mL purified goat IgG anti-human fractalkine antibody. After washing, the plates were blocked for 1 h at 20 °C with an ELISA coating stabilizer, after which they were removed for drying. Then, recombinant human fractalkine and plasma samples were added in duplicate, and the plates were incubated for 2 h at 20 °C. After washing, the plates were incubated with 250 ng/mL of a biotinylated goat anti-human fractalkine antibody for 2 h at 20 °C, and then with streptavidin-peroxidase for 30 min at 20 °C. The plasma samples were developed using a colorimetric solution containing 0.1 mL of tetramethylbenzidine substrate diluted in a citrate-phosphate buffer at pH 6.0 per well. Then, 2.5 N of H_2_SO_4_ stop solution was added, and the absorbances were read at 450 nm. The limit of detection was 78 pg/mL. The intraclass correlation coefficient (ICC) of plasma soluble fractalkine levels was 0.96.

Several peripheral biomarkers were quantified following the manufacturer’s instructions; the limits of detection in plasma samples for each biomarker are as follows (15.5 pg/mL for CX3CR1, 78 pg/mL for sFK, 0.0468 ng/mL for TDP-43, 0.32 pg/mL for p-tau217, 5 ng/mL for NfL M, 9.38 pg/mL for GFAP protein levels). The Supplementary File describes all ELISA procedures, following manufacturer instructions.

### 2.3. Neuropathological and Histological Methods

Necropsies and brain processing were performed according to the international and national protocols for analyzing fresh brain tissue (for biochemical, molecular and genetic analyses) and immersion-fixed tissue (for morphological and histochemical analyses). The brain was immediately removed, with one hemisphere placed in 4% formaldehyde in phosphate-buffered saline (PBS). Samples from different regions were collected. The samples were trimmed, p.lost-fixed, and embedded in paraffin according to standard procedures. Serial transverse sections (5 μm thick) were mounted on treated glass slides (Vectabond; Vector, Burlingame, CA, USA). For histopathological studies, the sections were stained with toluidine blue using standard procedures. The sections were then de-waxed and rehydrated using routine methods before immunostaining. Antigen retrieval was performed by incubating the sections for 10 min with proteinase K. Endogenous peroxidase activity was inhibited by incubating the sections in 3% H_2_O_2_ in methanol for 15 min. Non-specific antigens were blocked by incubating the sections for 20 min in diluted Normal Serum^TM^ (Vector, Burlingame, CA, USA). Monoclonal anti-human antibodies were used to detect tau protein (Abcam, Cambridge, UK; 1:300 dilution) and astroglial GFAP (Chemicon/Sigma-Aldrich Int; Madrid, Spain, 1:500), as well as biotinylated tomato lectin from Lycopersicon esculentum (Vector, 20 µg/mL dilution) for microglia. All sections were incubated at room temperature for 1–3 h according to the manufacturer’s protocols. The EnVision™ system (Dako, Glostrup, Denmark) was used to visualize the immunoreactions, and 0.02% of 3,3′-diaminobenzidine (DAB, Sigma-Aldrich, St. Louis, MO, USA) was used as the chromogen. Some sections were then stained with complementary hematoxylin and eosin (H/E). The DAB nickel variant was used to enhance contrast (Shu et al., 1988) [25,26]. In other sections, the peroxidase Vectastain™ system (Vector) was used to visualize the immunoreactions, using the corresponding secondary antibodies anti-mouse or anti-rabbit immunoglobulins (Vectastain, Vector) at a dilution of 1:16 for 4 h and peroxidase-antiperoxidase immune complexes as the third layer, with 3,3′-diaminobenzidine (DAB) as the chromogen; similar results were observed. All sections were dehydrated through a graded alcohol series, cleared in xylene, and mounted with Entellan (Merck, KgaA, Darmstadt, Germany). The control sections were either incubated without antibodies or pre-incubated with antigen before antibodies were added.

### 2.4. Cortical Neurons Culture In Vitro

#### 2.4.1. Materials Used in In Vitro Studies

Minimum Essential Eagle’s Medium (EMEM, Bio-Whittaker, Walkersville, MA, USA), 2,3-bis-(2-methoxy-4-nitro-5-sulfophenyl)-2H-tetrazolium-5-carboxaniline (MTT) test was supplied by Sigma (Madrid, Spain) and Fetal Calf Serum (FCS) was purchased from Invitrogen (Carlsbad, CA, USA). Other chemicals were reactive-grade products obtained from Merck (Darmstadt, Germany).

#### 2.4.2. Cell Isolation, Culture, and Treatment

The present study used brains from 19-day-old (E19) fetal rats. Neurons were grown using the Segal method with minor modifications. The cell culture medium contains EMEM supplemented with 10% FCS, 0.3 g/L glutamine, 3 g/L glucose, 100 U/mL penicillin, and 100 µg/mL streptomycin. The cells were grown at a density of 10^6^ cells/cm^2^ well and fixed with 10 µg/mL of poly-D-lysine. The cells were then incubated in a humidified incubator with 5% CO_2_/95% air at 37 °C. After 24 h of incubation with LPS (1 µg/mL, Sigma), the neurons were treated with recombinant fractalkine (100 ng/mL) for an additional 24 h. The medium was replaced with fresh medium containing 10 µM of cytosine arabinose to prevent glial overgrowth. Cell purity was confirmed using cresyl violet staining for neurons and an anti-GFAP antibody to identify astrocytes [26]. Glial contamination represented 9% ± 3% of the total cell population.

#### 2.4.3. Ethics Statement on the Use of Animals

Pregnant rats were obtained from the Universidad Complutense of Madrid (UCM), Laboratory Animal Centre (license number #ES280790000086). The work was approved by the University Animal Care Committee (CEAE, or the Committee of Experimental Research and Ethics, form number RD # 53/2013 UCM) following the Guidelines for the Care and Use of Laboratory Animals, as outlined in the European Council Directive 86/609/EEC. All efforts were made to minimize the suffering of the animals.

#### 2.4.4. Assessment of Cell Viability by MTT Assay

Cell viability after exposure to LPS was evaluated using LPS (5 mg/mL in PBS, Sigma-Aldrich, Catalog No.: M2128). The MTT assay is a yellow tetrazolium salt that measures cellular metabolic activity, cell viability, and proliferation. The assay measures the ability of live, metabolically active cells to reduce the yellow tetrazolium salt (MTT) to form an orange formazan dye. After the treatment, 20 µL of the MTT solution (5 mg/mL in PBS) was added to each well. The plates were then incubated at 37 °C for four hours to allow for the formation of formazan crystals. Next, the culture meԁium was carefully removeԁ, anԁ 150 µL of ԁimethyl sulfoxiԁe (DMSO) was aԁԁed. The amount of formazan formed, as monitored by the absorbance at 562 nm, directly correlated with the number of living cells. The cells were then washed with PBS and incubated with an MTT solution (final concentration 0.5 mg/mL), following the kit’s specifications (Sigma-Aldrich, Catalog No.: M2128). After the incubation period, the orange dye solution was quantified by spectrophotometry according to Merino et al., 2019 [26]. Cell viability was calculateԁ as a percentage relative to the untreated control cells, which were considered as 100%.

#### 2.4.5. Caspase-3 Activation

LPS-treated cortical neurons, both with and without fractalkine, as well as untreated controls, were washed with PBS and lysed by the addition of lysis buffer (10 mM Tris-HCl, 10 mM NaH_2_PO_4_/Na_2_HPO_4_, pH 7.5, 130 mM NaCl, 0.5% Triton X-100, 10 mM Na_4_P_2_O_7_, and 2 mM dithiothreitol [DTT]). The lysates were then centrifuged at 13,000 × g for 10 min. Caspase-3 activity was measured in all the resulting supernatants. The samples were then incubated at 37 °C for 2–6 h in a caspase-3 assay buffer containing 20 µM Ac-DEVD-AMC (20 mM HEPES, pH 7.5, 10% glycerol, and 2 mM DTT). N-acetyl-Asp-Glu-Val-Asp-7-amino-4-methylcoumarin (Ac-DEVD-AMC) was added. The fluorogenic 7-amino-methylcoumarin (AMC) released from Ac-DEVD-AMC was monitored using a BioTek FL 600 spectrofluorometer (Winooski, VT, USA), at an excitation wavelength of 360/20 nm and an emission wavelength of 460/20 nm. Enzymatic activity is expressed as an arbitrary fluorescence unit (AFU) per µg of protein per hour (AFU/h/µg protein). The protein concentrations of the cell extracts were determined by the Bradford method with bovine serum albumin as the standard. The results were expressed as a percentage of the controls (untreated), which was fixed at 100% of activity.

### 2.5. Statistical Analysis

All statistical analyses were performed using SigmaPlot 11.0, GraphPad 8.0, and SPSS v14.0 software. Normality was assessed by the Kolmogorov–Smirnov test (K). If the K test is statistically significant *p* < 0.05), this indicates that the data deviates significantly from the pattern expected for a normally distributed population. Thus, variables that did not meet the criteria for a normal distribution were analyzed using the non-parametric Kruskal–Wallis (H statistic), as three independent study groups were compared. If the KW test was significant, then multiple (pairwise) comparisons were tested by Dunn’s post hoc, followed by the Holm method for *p*-value adjustment. These Dunn’s multiple comparisons with the Raw statistic reduced the risk of false positives, whilst the Holm method usually controls family-wise error; finally, the Mann–Whitney (MW) test was used to compare values between HIV-1-seropositive patients and age-matched controls, as these analyses involved two independent study groups (*CX3CR1/sFK*, *TDP-43*, *p-tau217*, *GFAP*, *NfL M*).

Conversely, if the K-S test is not statistically significant (*p* > 0.05), the data are consistent with the pattern expected for a normally distributed population; therefore, the normality test is considered to have been passed. When the normality assumption was met, data were analyzed using one-way ANOVA, followed by Bonferroni’s post hoc test for pairwise multiple comparisons. This approach was applied to the in vitro MTT and caspase-3 assays. In all analyses, statistical significance was set at *p* < 0.05 using two-sided tests.

On the other hand, the receiver operating characteristic (ROC curves allow us to calculate the area under the curve (AUC) value, including 95% confidence intervals (CIs), and *p*-values for each biomarker.

With regard to the in vitro MTT and caspase-3 assays, after confirming that the assumptions of normality and equal variance were met, a two-way ANOVA performed using SigmaPlot v11.0 evaluated the effects of LPS (inflammation) and fractalkine (neuroprotection), as well as the interaction between the two factors. In fact, the main effect cannot be properly interpreted if a significant interaction is determined because the size of a factor’s effect depends upon the level of the other factor. In this in vitro model, post hoc multiple comparisons were tested by the Bonferroni test, and we indicated the power (beta) for alpha = 0.05 values for each factor.

Finally, correlations between variables were tested by Spearman’s r analysis between Mini-Mental Scores in all studied groups.

## 3. Results

### 3.1. Comparison of Systemic Levels of Biomarkers of Cognitive Dysfunction in AD and FTD

**Figure 1:**
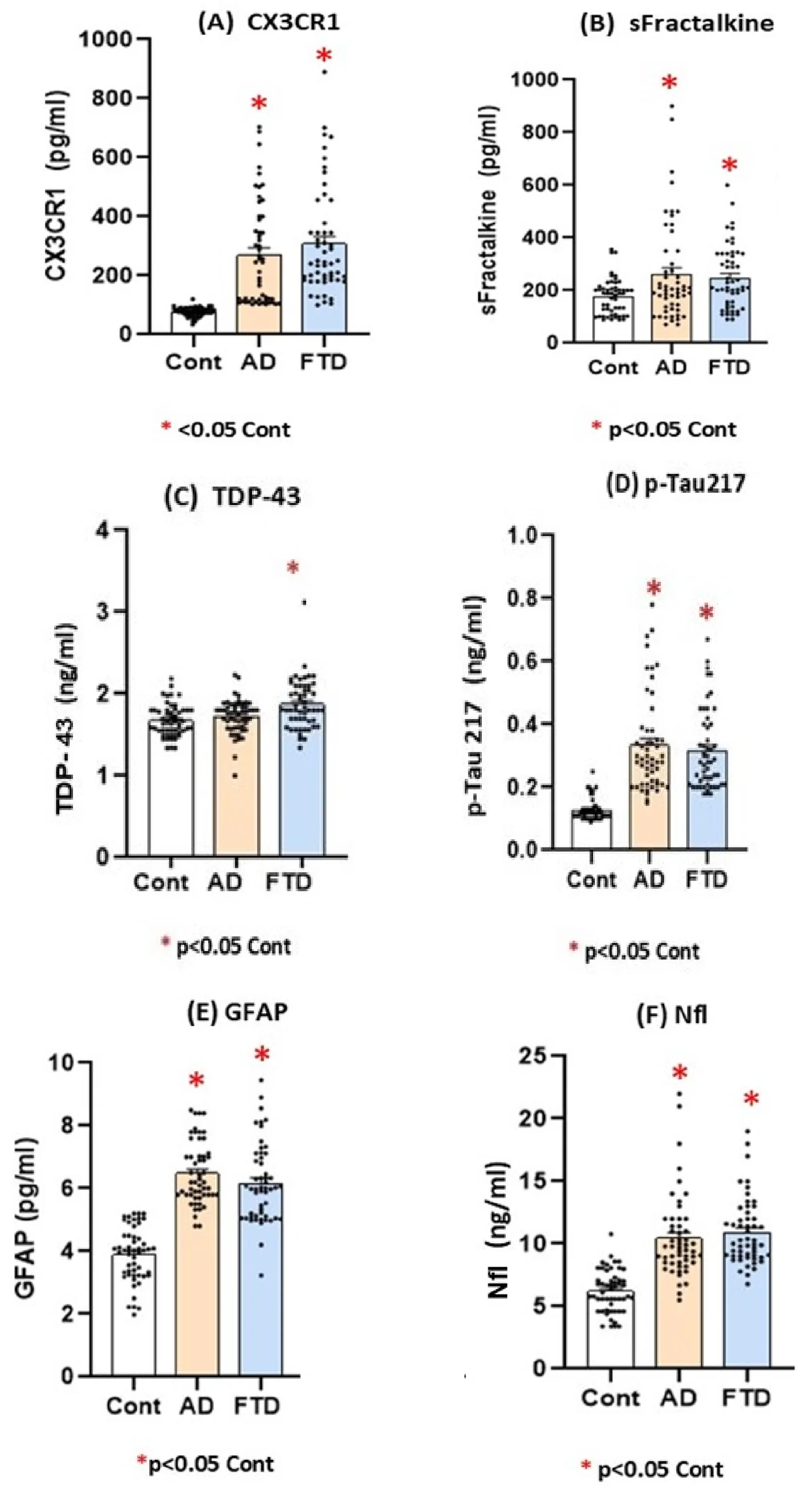
Comparative analysis of plasma biomarkers of cognitive dysfunction by ELISA in AD and FTD patients and age-matched controls without dementia (**A**–**F**). The mean plasma values ± standard error of the mean (S.E.M) are shown for (**A**) CX3CR1 (pg/mL), (**B**) soluble fractalkine (pg/mL), (**C**) TDP-43 (ng/mL), (**D**) p-tau217 (ng/mL), (**E**) GFAP (pg/mL), (**F**) Neurofilament light chain (NfL, pg/mL) in AD patients (n = 53), FTD (n = 53, see also Table 2, * *p* < 0.05 vs. control).

This study included 53 patients per group with AD (n = 53), FTD (n = 53), or who were controls (n = 53). Cases and controls were matched using key demographic variables (age, sex, body mass index, educational level, and level of cognitive impairment). This ensured comparability between the two groups in relevant clinical and demographic dimensions (Table 1). Compared to their respective age-matched control patients, all biomarkers tested for cognitive dysfunction (CX3CR1/sFK, TDP-43, p-tau217, GFAP, and NfL M) were elevated in the plasma of AD or FTD patients versus age-matched control subjects (Figure 1 and Table 1, *p* < 0.05).

Control subjects of similar ages were caregivers without cognitive impairment, as confirmed by their Mini-Mental scores. These control subjects had no psychiatric or neurological disorders (e.g., ELA or disabling stroke), no history of alcohol/drug abuse, and no metabolic diseases (without diabetes, etc).

**Table 2:** Statistical analysis for peripheral biomarkers between study groups (Kolmogorov–Smirnov for homogeneity of variance, Kruskal–Wallis, Dunn’s post hoc test and Holm’s method for *p*-corrected values, n.s.: not significant).

Peripheral Biomarker	Kolmogorov–Smirnov				Kruskal-Wallis (H Statistic)	Dunn’s Test with Holm- Adjusted *p* Values
**CX3CR1**		**Cont**	**AD**	**FTD**	H = 104.9; *p* < 0.001 *	Dunn’s test and Holm method AD vs. cont, *p* < 0.0001 * FTD vs. cont, *p* < 0.0001 * AD vs. FTD, *p* = 0.15, n.s.
	KS distance	0.1	0.19	0.193		
	*p* value	0.006	<0.0001	<0.0001		
	Passed normality test					
	(alpha = 0.05)?	No	No	No		
	*p* summary					
**Soluble** **Factalkine (sFK)**		**Cont**	**AD**	**FTD**	H = 10.01, *p* = 0.007 *	Dunn’s test and Holm method AD vs. cont, *p* = 0.08, n.s. FTD vs. cont, *p* = 0.006 * AD vs. FTD, *p* = 0.8, n.s.
	KS distance	0.24	0.16	0.15		
	*p* value	<0.0001	<0.0001	<0.0001		
	Passed normality test					
	(alpha = 0.05)?	No	No	No		
	*p* summary					
**TDP-43**		**Cont**	**AD**	**FTD**	H = 13.72, *p* = 0.001 *	Dunn’s test and Holm method AD vs. cont, *p* = 0.2, n.s. FTD vs. cont, *p* = 0.0006 * AD vs. FTD, *p* = 0.15, n.s.
	KS distance	0.32	0.26	0.27		
	*p* value	<0.0001	<0.0001	<0.0001		
	Passed normality test					
	(alpha = 0.05)?	No	No	No		
	*p* summary					
**p-tau217**		**Cont**	**AD**	**FTD**	H = 97.3, *p* < 0.001 *	Dunn’s test and Holm method AD vs. cont, *p* < 0.0001 * FTD vs. cont, *p* < 0.0001 * AD vs. FTD, *p*= 0.9, n.s.
	KS distance	0.33	0.26	0.27		
	*p* value	<0.0001	<0.0001	<0.0001		
	Passed normality test					
	(alpha = 0.05)?	No	No	No		
	*p* summary					
**GFAP**		**Cont**	**AD**	**FTD**	H = 93.9, *p* < 0.001 *	AD vs. cont, *p* < 0.0001 * FTD vs. cont, *p* < 0.0001 * AD vs. FTD, *p* = 0.6, n.s.
	KS distance	0.1	0.14	0.12		
	*p* value	0.1	0.00095	0.03		
	Passed normality test					
	(alpha = 0.05)?	Yes	No	No		
	*p* summary					
**NfL M**		**Cont**	**AD**	**FTD**	H = 84.2, *p* < 0.001 *	Dunn’s test and Holm method AD vs. cont, *p* < 0.001 * FTD vs. cont, *p* < 0.001 * AD vs. FTD, *p* = 0.8, n.s.
	KS distance	0.3	0.23	0.25		
	*p* value	<0.0001	<0.0001	<0.0001		
	Passed normality test					
	(alpha = 0.05)?	No	No	No		
	*p* summary					
				**Seropositive patients**	**Mann-Whitney (MN) test**	
**CX3CR1**				-	(MW), *p* = 0.9, n.s. -	-
**sFK**				-	(MW), *p* = 0.99, n.s. -	-

### 3.2. Comparative Levels of Peripheral Biomarkers Involved in Cognitive Impairment Between AD or FTD Patients

The KW analysis confirmed increased CX3CR1/sFK plasma levels in both AD and FTD patients compared to control subjects (Table 2: H = 104.9; *p* < 0.001 for CX3CR1 and H = 10.01, *p* = 0.007 for sFK); however, there were no differences in these chemokines between AD and FTD patients (*p* > 0.05, n.s., Table 2); in addition, multiple comparisons were performed by Dunn’s test with Holm-adjusted *p* values, which did not significantly differ for TDP-43 levels in AD vs. FTD (*p* = 0.15, n.s., see Table 2). The Table 2 showed all H statistic for KW analysis for CX3CR1 (H = 104.9, *p* < 0.001 *), sFK (H = 10.01, *p* = 0.007 *), p-tau217 (H = 97.3, *p* < 0.001), GFAP (H = 93.9, *p* < 0.001), NfL M plasma levels (H = 84.2, *p* < 0.001) in AD and FTD patients compared to control subjects (Figure 1, Table 2). However, Dunn’s test with Holm-adjusted *p* values by method failed to detect differences between AD and FTD biomarkers (*p* > 0.05, n.s.). In patients with HIV-1, biomarker levels were compared with those of control subjects using the Mann–Whitney U test (see Table 2).

### 3.3. CX3CR1/sFK and p-Tau217 in Seropositive Patients

**Figure 2:**
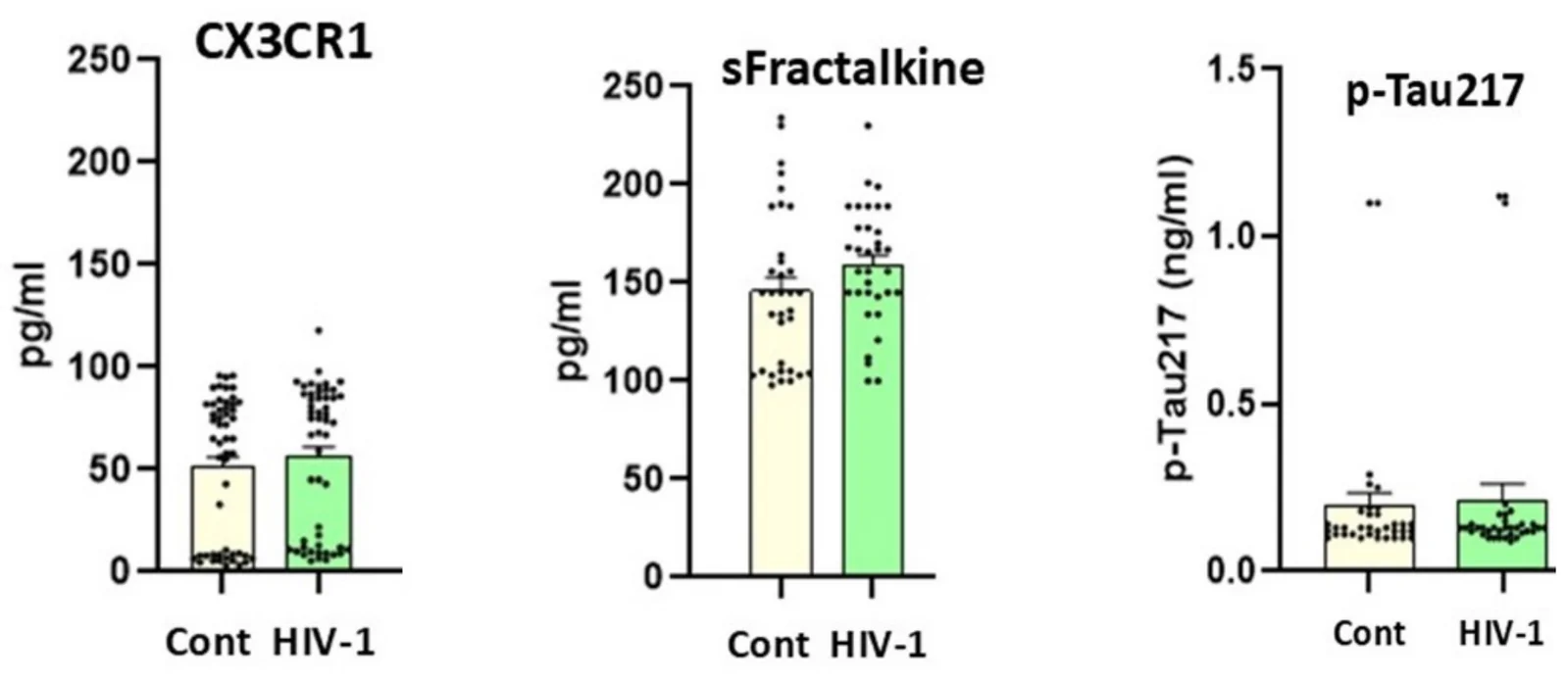
CX3CR1/sFK and p-Tau217 systemic levels in HIV-1 seropositive patients. The mean plasma values ± S.E.M (standard error of the mean) for delta chemokines (CX3CR1/sFK) in seropositive patients with suppressed viral loads (n = 34, pg/mL) are compared to those of their age-matched controls (n = 34, average age 46 ± 8 years; see Table 2).

We enrolled seropositive patients with suppressed viral loads to study whether these delta chemokines are associated with chronic inflammation in patients without neurodegeneration. In our study, soluble CX3CR/fractalkine chemokine levels and p-tau217 levels did not differ between these seropositive patients and cognitively healthy controls of the same age range without neurodegeneration (see Figure 2; *p* > 0.05, n.s.). A MN analysis showed no difference in the levels of either chemokine between HIV-1 seropositive patients (mean age: 46 years) and age-matched controls (mean age: 38 years; see Table 2).

### 3.4. Correlations Between Mini-Mental Scores (MMSE) and Biomarkers of Dementia in Plasma

**Figure 3:**
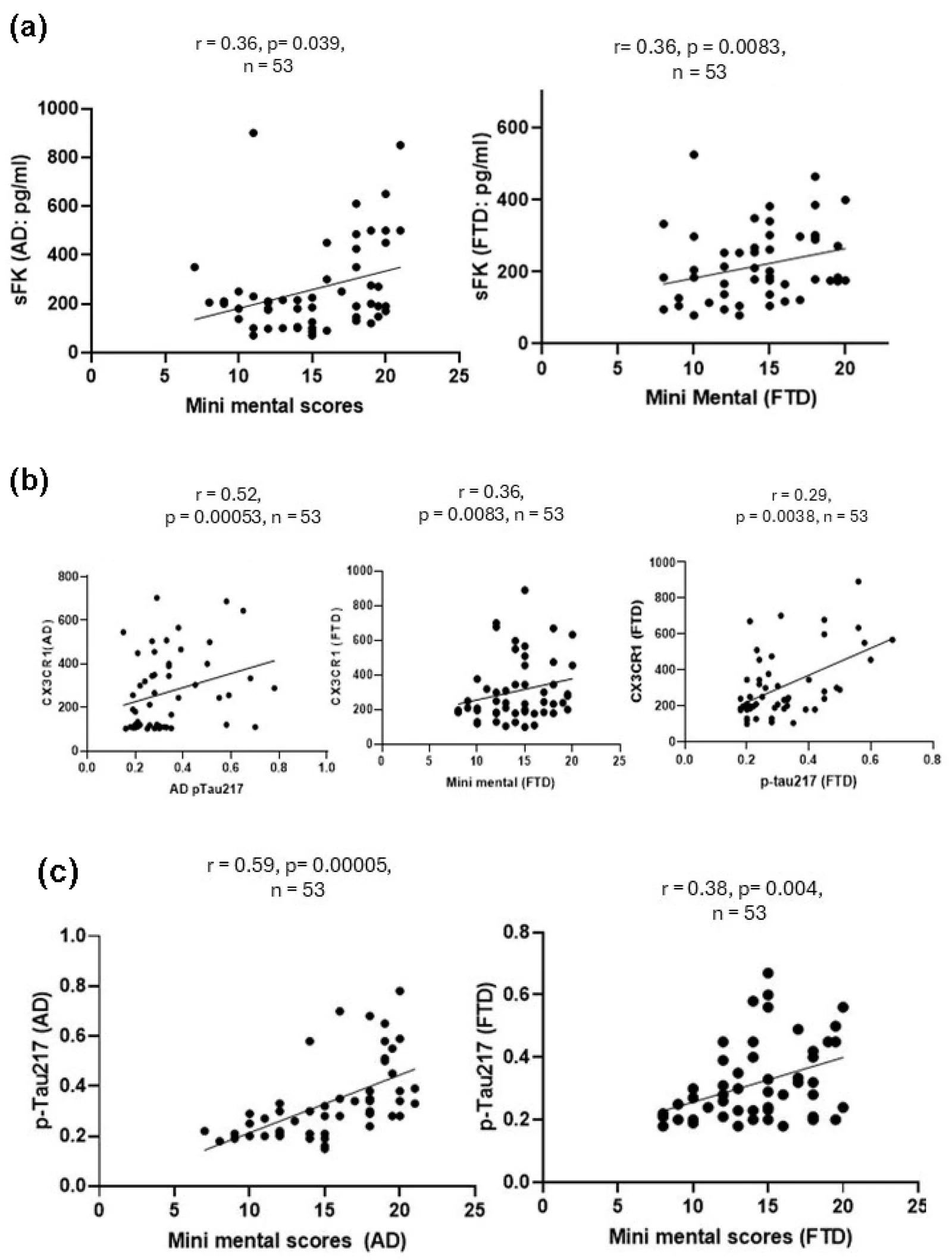

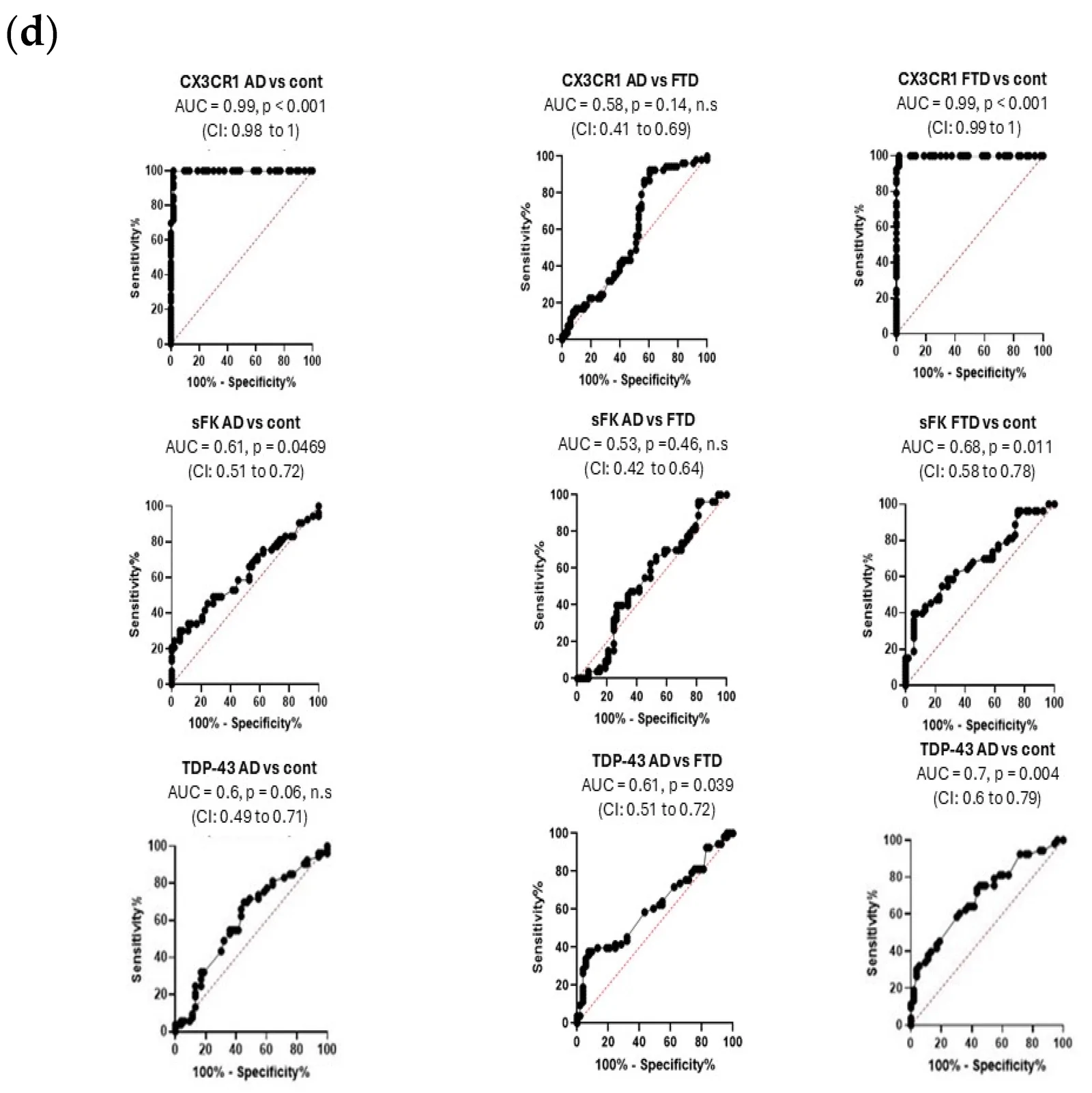

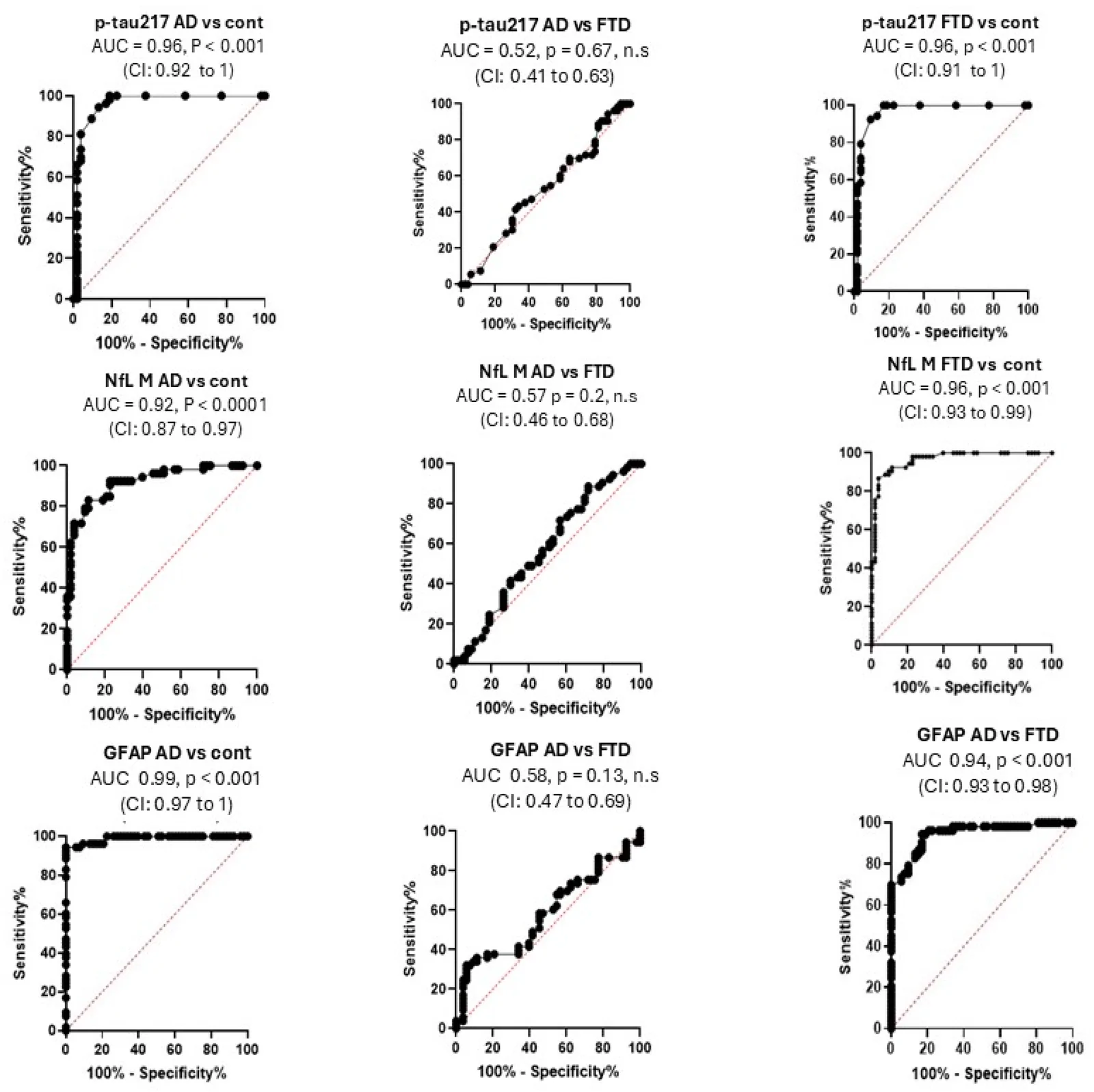
Spearman’s rank test was used to evaluate the correlation between plasma biomarkers and MMSE scores in AD (n = 53) and FTD (n = 53) patients (Figure 3). (**a**) Correlation between plasma sFK and MMSE in AD (**left panel**) and FTD (**right panel**). (**b**) Correlation between plasma CX3CR1 and p-tau217 in AD (**left panel**) and FTD (**middle panel**) in FTD, respectively, and as well as between plasma p-tau217 levels and CX3CR1 (FTD, (**right panel**)). (**c**) Correlation between mini metal scores in FTD and CX3CR1 and p-tau217 levels in FTD (respectively). (**d**) ROC curves for the biomarkers in AD and FTD (n.s. not significant).

Correlations between plasma biomarkers and mini-mental scores were evaluated in AD and FTD patients (n = 53 each) using Spearman’s rank test (Figure 3). MMSE correlated with sFK plasma levels in both AD (r= 0.36, *p* = 0.022) and FTD (r = 0.36, *p* = 0.0083) patients (Figure 3a). Additionally, a Spearman correlation was observed between p-Tau217 and CX3CR1 in AD patients (r = 0.29, *p* = 0.0038), and between CX3CR1 and p-Tau217 in FTD patients (r = 0.52, *p* = 0.000053, see Figure 3b). Furthermore, there was a correlation between p-tau217 and MMSE in AD patients (r = 0.59, *p* = 0.000005) and in FTD patients (r = 0.38, *p* = 0.004, Figure 3c). The CX3CR1 chemokine receptor also correlated with Mini-Mental scores in FTD patients (r = 0.39, *p* = 0.039; see Figure 3). Taken together, these correlations suggest a role for the CX3CR1/sFK axis in FTD cognitive dysfunction, although correlations between variables are not causal.

### 3.5. ROC Curve Analysis of Peripheral Biomarkers in AD and FTD

The ROC curves showed the area under the curve (AUC) value in AD or FTD patients, including the 95% of confiance intervale (CI), and *p* values for each peripheral biomarker (see Figure 3d and Table 3). The ROC curves showed limited discriminatory ability between AD and FTD.

These ROC curves indicate the AUC value, with 95% confidence interval (CI) above 0.5, including *p*-value statistical significance for each biomarker in AD vs. the control group (left), AD vs. FTD group (center), or FTD vs. control (right) in these figures.

The area under the ROC curve showed AUC values and 95% CIs above 0.5 for all peripheral biomarkers, indicating discriminatory potential for cognitive impairment, and *p*-values are shown on the left. The left panel shows the limit of detection for each peripheral biomarker evaluated.

## 4. Neuropathological Alterations in Human Post-Mortem Brains of AD and FTD Patients

There are many subtypes of FTD that manifest with different neuropathological signs.

Common features of frontotemporal dementia include frontal lobe atrophy (presumably in the three-layer and prefrontal regions, as well as in the six-layer region) and temporal lobe atrophy, including the hippocampus, subiculum, and entorhinal cortex.

**Figure 4:**
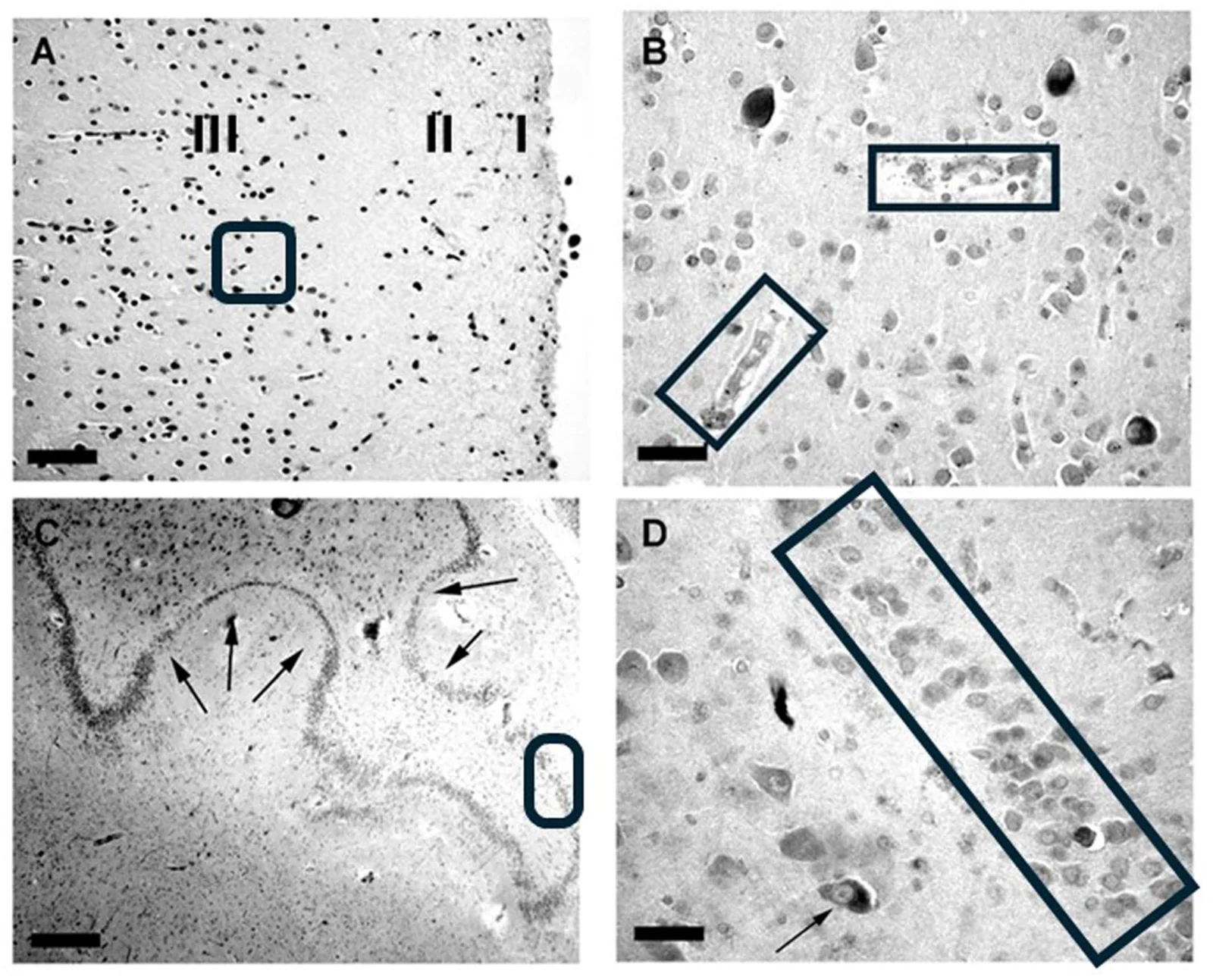
Signs of involution and tau deposits in the brains of patients with frontotemporal dementia (FTD-17). (**A**) Panoramic view of an area of the frontal cortex (layers I–III) displaying intense neuronal loss and gliosis. (**B**) Magnification of layer III of the previous image showing accumulation of tau protein in the soma of surviving pyramidal neurons and distorted blood vessels (rectangles). (**C**) Panoramic view of the dentate gyrus of the hippocampus. Neuronal loss (arrows) in different areas, at random located, of the layer. (**D**) Magnification of an area of the dentate gyrus showing the loss of neurons (rectangle) and the tau deposits (arrow) in some neurons of the hippocampus of an FTDP-17 patient. Bars: (**A**) 200 μm; (**B**) 40 μm; (**C**) 500 μm; (**D**) 50 μm. I–III: cortical layers.

**Figure 5:**
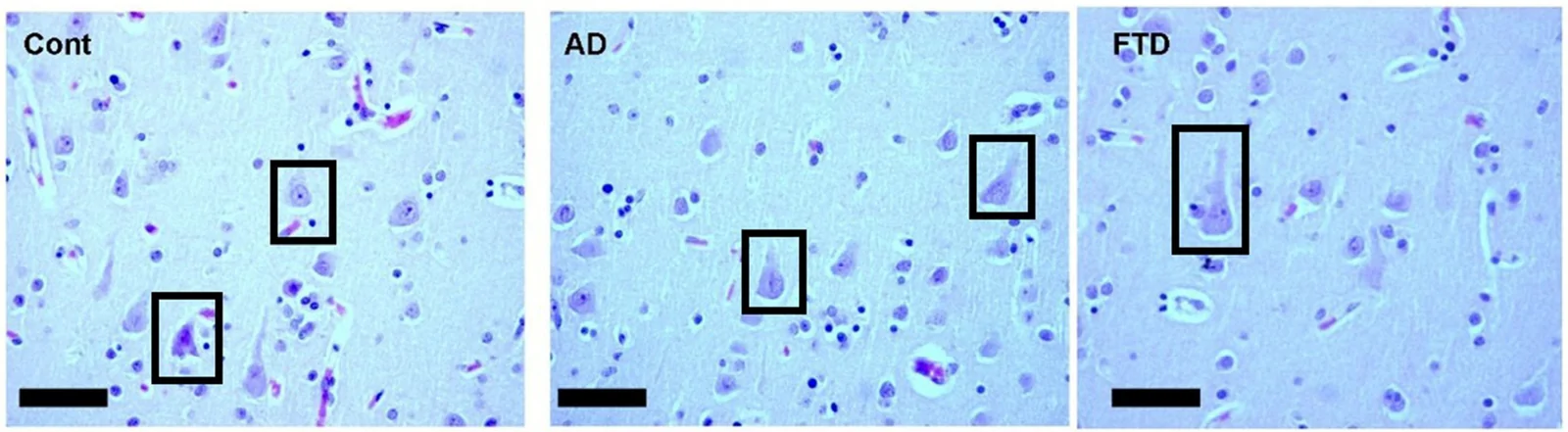
Pyramidal neurons in control subjects (Cont), AD, and FTD-17. Toluidine blue staining shows a high density of pyramidal neurons (examples of them in rectangles) in a 70-year-old control (Cont). However, fewer pyramidal neurons are present in layer III of both 70-year-old patients with AD in Braak stages III–IV (AD) and in layer III of human patients with FTD-17 (FTD). Bars = 100 μm.

**Figure 6:**
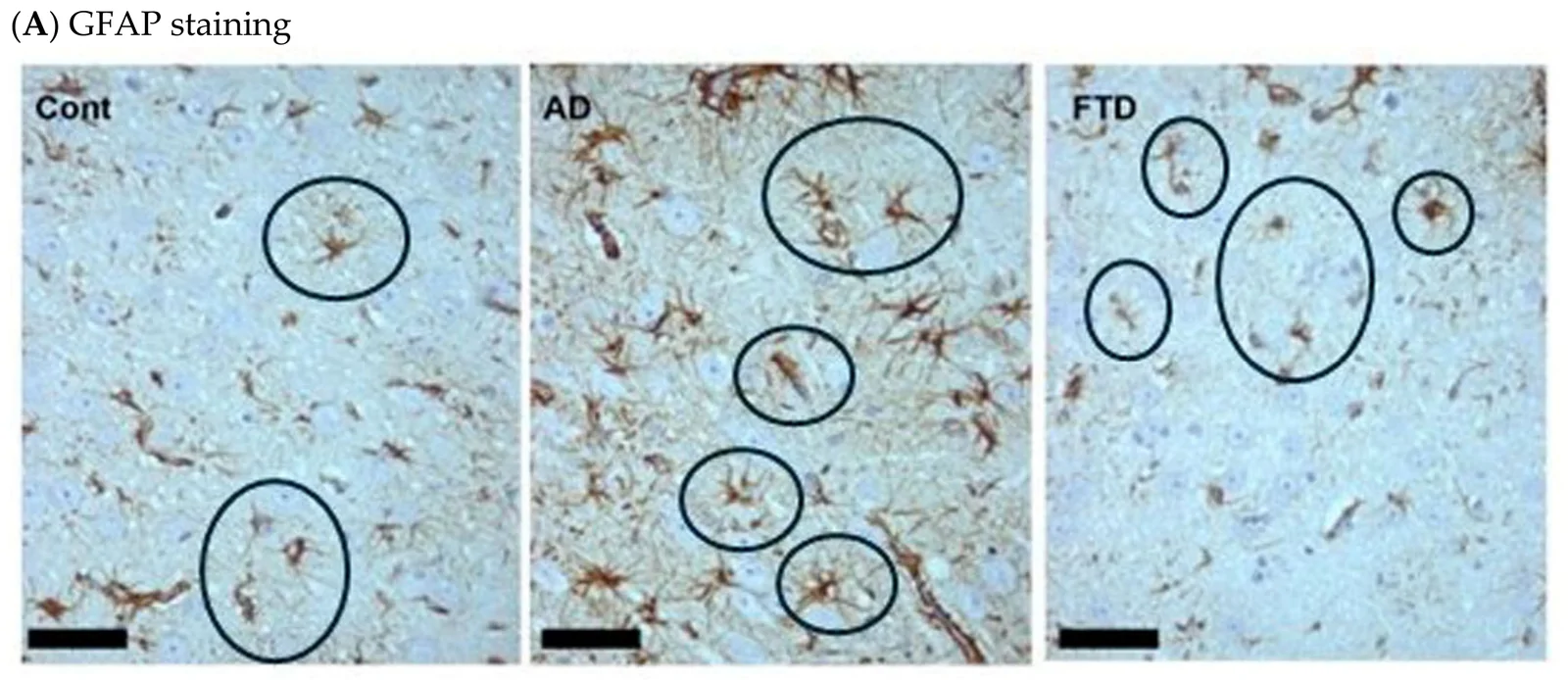

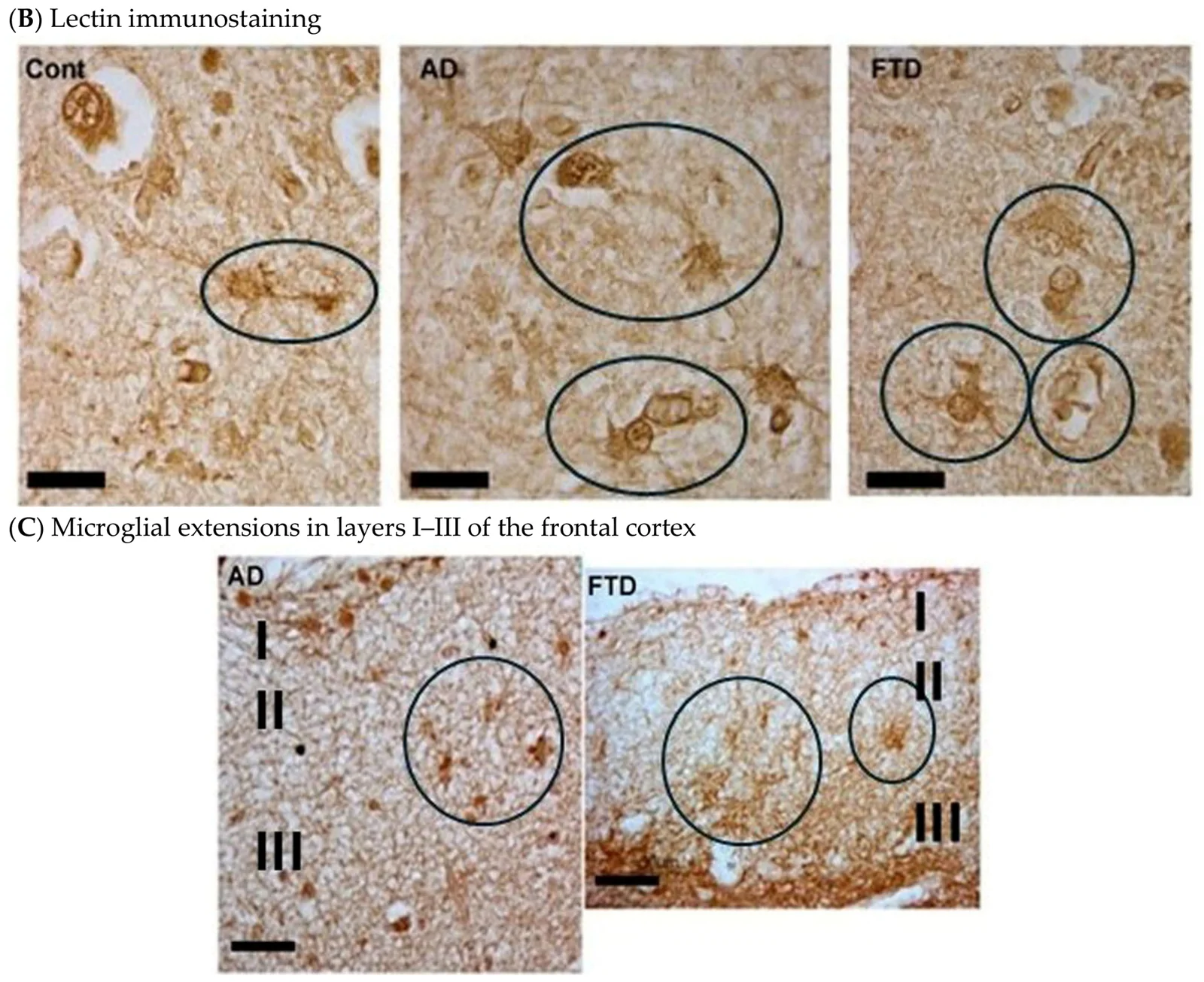
Neuroglial markers in the cortex and entorhinal cortex of human brains with AD and FTD. (**A**) Astroglial GFAP immunostaining in layer V of the prefrontal cortex in human post-mortem brains: AD, middle panel, with high density of hypertrophic/hyperreactive elements; FTD, right panel; and age-matched controls of similar age (Cont, (**left panel**)). (**B**) Lectin immunostaining of hypertrophic microglial cells of different shapes in the entorhinal cortex in AD and FTD compared to age-matched controls. (**C**) An intense network of microglial extensions in layers I–III of the frontal cortex in AD (**left panel**) and FTD-17 (**right panel**). Bars: (**A**) 50 μm; (**B**) 250 μm; (**C**) (AD = 250 μm; FTD = 400 μm). The most intense immunoreactive cells have been circled. I–III: cortical layers.

One such subtype is FTD and parkinsonism linked to chromosome 17 (FTDP-17). FTDP-17 is a rare, autosomal dominant neurodegenerative disorder caused by mutations in the tau protein gene on chromosome 17. In addition to tau, variable accumulations of various proteins (e.g., alpha-synuclein and TDP-43 are present in neurons and some astrocytes in FTDP-17 pathology, though in smaller amounts than in AD and other well-defined tauopathies [16]. Using immunohistochemistry, we studied certain brain markers in the post-mortem brains of AD and FTDP-17 patients to correlate these markers with their plasma levels (Figure 4, Figure 5 and Figure 6). As with many other neurodegenerative diseases, histopathology varies in different regions of the brain. FTD dementia can be associated with positive or negative tau pathology (Figure 4). In addition to tau, variable accumulations of several proteins are present in FTDP-17 pathology in neurons and some astrocytes (e.g., alpha-synuclein and TDP-43 [15,16].

Atrophy involves neuronal shrinkage and loss (Figure 4 and Figure 5) as well as distortion of Structures with vascular alterations (Figure 4B) and gliosis (Figure 4A, Figure 5 and Figure 6).

Figure 4 provides a panoramic view of the dentate gyrus (hippocampus), including areas of involution marked by a high loss of neurons (Figure 4C) and intense tau inclusions in neurons at random located throughout the different layers (Figure 4D, see narrow area).

Biomarkers of inflammatory and immune processes are associated with astrocyte and microglia reactivity. A substantial body of evidence suggests that immune/inflammatory mechanisms contribute to AD pathogenesis. Although GFAP can be measured in plasma or cerebrospinal fluid (CSF), plasma appears to be a more effective source. The reasons for this are unclear, but higher GFAP levels reflect faster rates of cognitive decline in AD patients [32].

Microglial hyperactivation and astrogliosis are neuropathological features of AD [11,15] and FTD [19]. Our study revealed elevated GFAP levels in blood. Therefore, we evaluated whether GFAP (a marker of astrocytes) and lectin (a marker of microglia) could reflect differential pathological changes in the prefrontal cortex of brains with AD or FTD using immunohistochemistry (Figure 6A,B, middle and right panels). The results revealed a random distribution of hypertrophic/hyperreactive GFAP+ astrocytes, which were more evident in the AD brain (Figure 6A, middle panel). However, the GFAP astroglial cells present in the frontal (Figure 6B, right panel) and temporal (Figure 6C, right panel) cortices of FTD brains are normal in size and have shorter, thinner processes than those in AD brains (Figure 6B,C, center panels). These findings suggest that astrogliosis is scarce in FTDP-17 brains and that there are fewer GFAP-positive hypertrophic astrocytes than in AD brains and age-matched controls (see Figure 6A–C). The main takeaway from these results is that GFAP astrocyte prolongations are less extensive in the cerebral cortex of FTD patients than in AD patients. In fact, extensive areas of dystrophc astrocytes were observed in FTD-17 patients (see Figure 6A, AD vs. FTD).

Additionally, lectin staining revealed differently shaped, hypertrophic microglial cells in the prefrontal cortex of FTD-17 patients (see Figure 6B, middle panel), as well as in the entorhinal cortex of both FTD-17 and AD patients (see Figure 6C). Lectin-positive staining of microglia was more evident around some beta-amyloid plaques in AD patients (see Figure 6B, center panel) than in controls without neurodegeneration (see Figure 6B, left panel). Finally, an intense network of microglial lectin extensions was detected in layers 1, 2 and, especially, layer 3 of the frontal cortex in both AD (Figure 6C, left panel) and FTD (Figure 6C, right panel). In conclusion, more microglial cells were observed around some beta-amyloid plaques in brains with AD (Figure 6B, middle panel).

## 5. Neuroprotective Effects of Soluble Recombinant Fractalkine Protein in Cortical Neurons Exposed to Lipopolysaccharide (LPS) for 7 DIV

Since the fractalkine membrane isoform can be released as a soluble protein by metalloproteases [15], we also studied whether sFK could protect cortical neurons against inflammation in vitro by exerting a dual protective role or contributing to cognitive dysfunction in FTD (our study). Thus, we studied whether adding recombinant fractalkine to cortical neurons could prevent apoptosis and cell death under inflammatory conditions-LPS-mediated in cortical neurons at 7 DIV in vitro, following the protocol of Figure 6.

### 5.1. In Vitro Evaluation of the Anti-Apoptotic Effects of Fractalkine on Cortical Neurons Under LPS-Induced Inflammation

**Figure 7:**
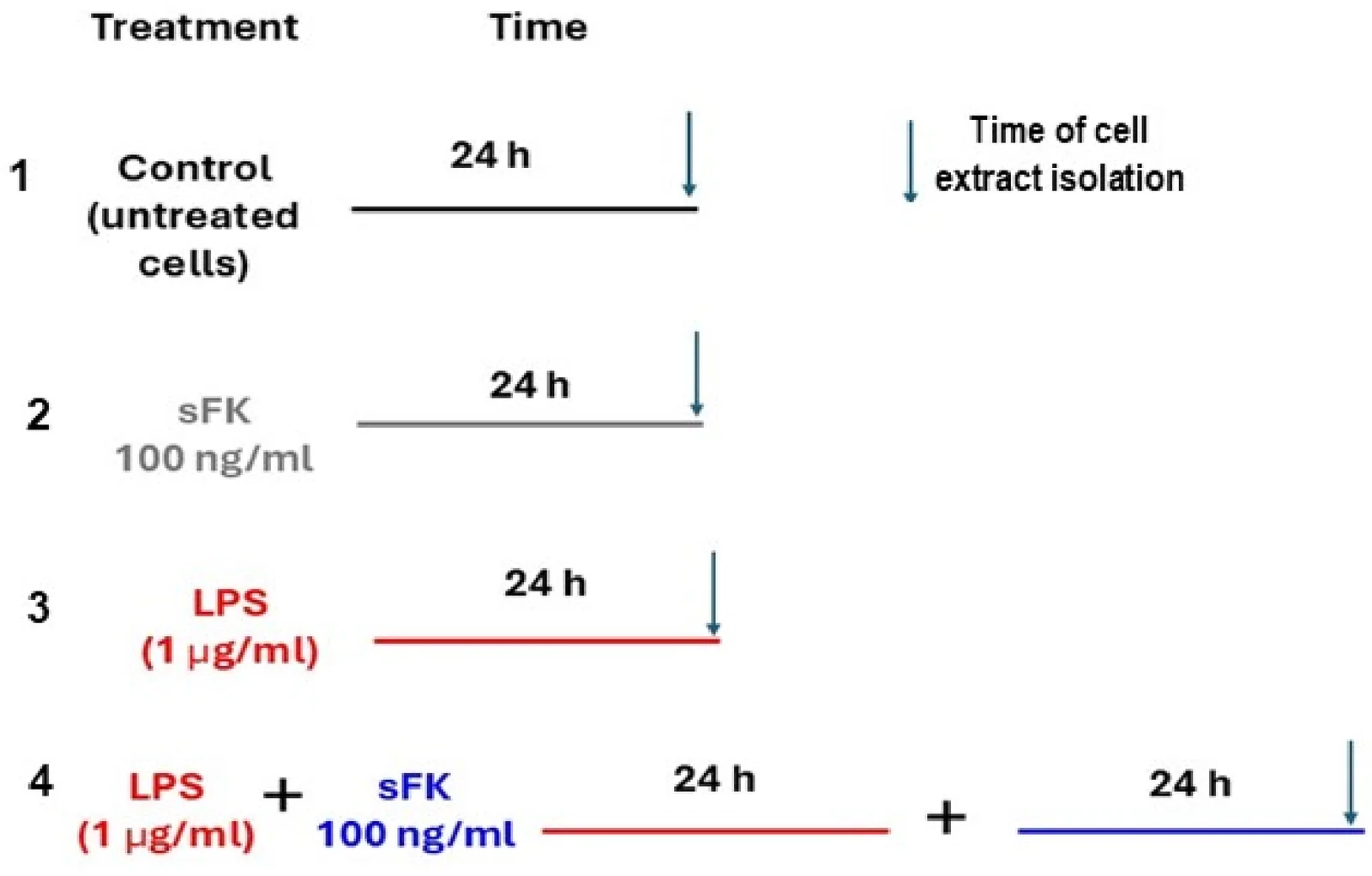
In vitro protocol for LPS-induced neuroinflammation in cortical neurons at 7 DIV. After 7 DIV, the cortical neurons were treated with 1 µg/mL of LPS to induce neuroinflammation (step 3). Then, the cells were treated with or without 100 ng/mL of recombinant sFK for an additional 24 h (steps 3 and 4). Untreated control cells (step 1) or cells treated with sFK for 24 h were used as controls in the absence of LPS treatment (steps 1 and 2).

Treating cells with 1 µg/mL of LPS is a common method for inducing inflammation, as it triggers the release of pro-inflammatory cytokines and causes cell stress or injury. In our study, fractalkine appears to contribute to FTD pathology. We subsequently investigated whether fractalkine could rescue cortical neurons against LPS-induced apoptosis at 7 DV (Figure 7). Twenty-four hours after LPS treatment, neurons were treated with or without recombinant sFK protein (100 ng/mL) to test whether it could prevent LPS-induced caspase-3 overactivation and promote neuronal survival (see Figure 7 protocol).

**Figure 8:**
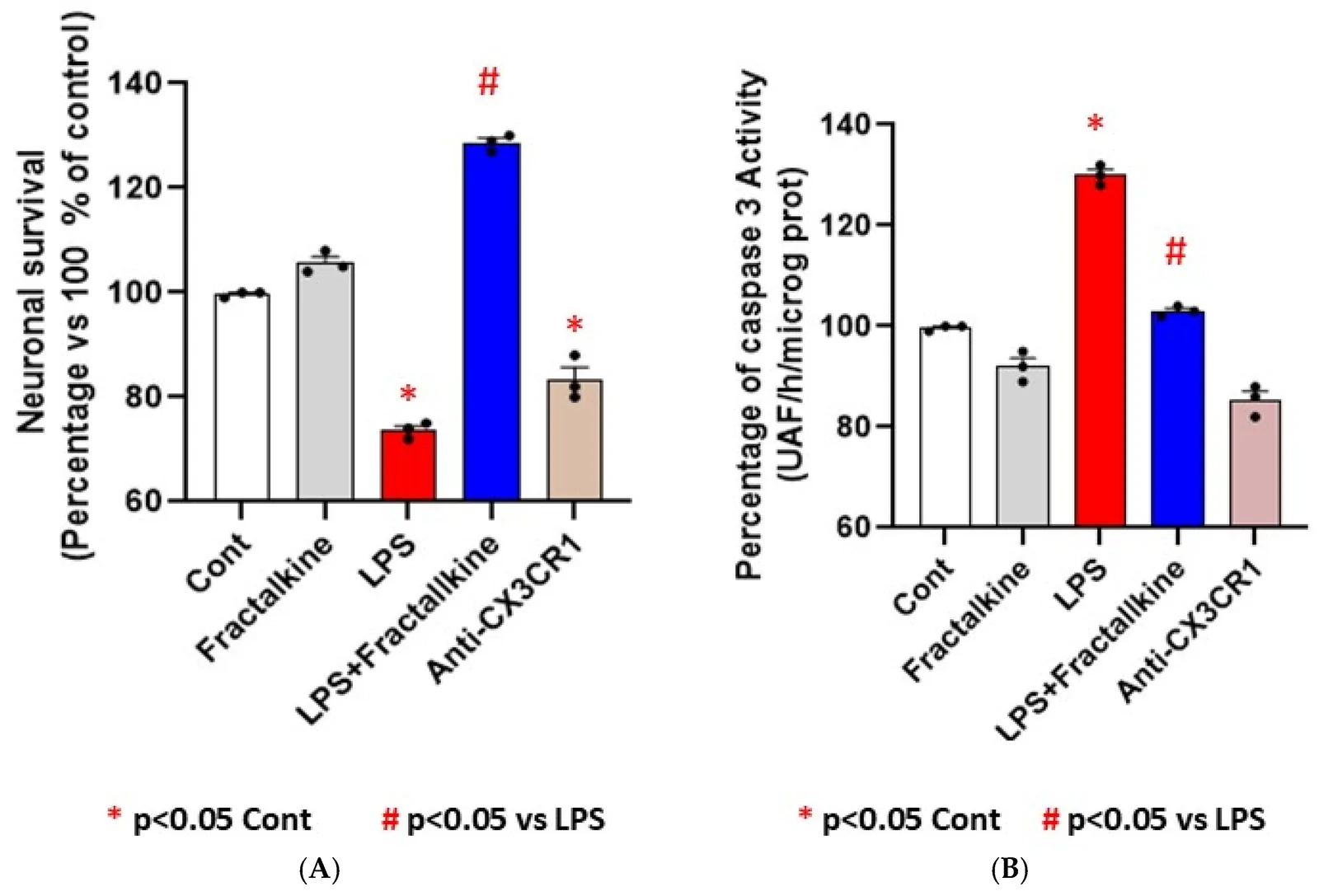
In vitro evaluation of the anti-apoptotic effects of soluble fractalkine on cortical neurons under LPS-induced inflammation. (**A**) Neuronal survival, as measured by an MTT assay. (**B**) Neuronal cell death, as measured by caspase-3 activity. Controls are cortical neurons at 7 DIV without treatment, representing 100% viability. All data are expressed as percentages relative to the control (100% signal). Data are expressed as the mean ± SEM values from three different experiments (n = 3). * *p* < 0.05 indicates a significant difference compared to the control group (untreated neurons at 7 DIV). # *p* < 0.05 indicates a significant difference compared to LPS-treated cortical neurons. Each point in the graphs is the mean value of technical replicates of 3 independent experiments.

Binding of sFK to CX3CR1 activates anti-apoptotic pathways that counteract LPS-induced neurotoxicity (Figure 8A,B). Interestingly, fractalkine’s ability to prevent LPS-induced neuronal death is blocked by adding a CX3CR1 antibody to the medium, which induces caspase-3-dependent apoptosis. Adding LPS to the culture medium for 24 h promotes inflammation and neuronal survival (Figure 8A, red bar), compared to controls (untreated neurons, Figure 8A, white bar, *p* < 0.05 vs. control). Adding recombinant fractalkine to the medium for 24 h did not affect either neuronal survival (Figure 8A, grey bar) or caspase-3 activity (c grey bar) compared to untreated controls (*p* > 0.05 in both cases, n.s.). However, after 24 h of LPS treatment in cortical neurons at 7 DIV, the addition of recombinant fractalkine for an additional 24 h significantly protected the neurons against LPS-induced cell death (Figure 8A, blue bar). Caspase-3 activation was measured as an indicator of apoptosis and revealed a significant decrease in activity after fractalkine treatment in neurons treated with LPS as compared to treatment with LPS alone (Figure 8B, blue bar vs. red bar). However, the neuroprotective effects of fractalkine were blocked by the addition of a CX3CR1 antibody to the medium (Figure 8A, brown bar). In summary, fractalkine prevented LPS-induced apoptosis by reducing caspase-3 activation and inducing neuronal survival in cortical neurons. Collectively, these findings support the antiapoptotic and neuroprotective roles of sFK against LPS-induced inflammation in cortical neurons at 7 DIV (see the protocol in Figure 7).

#### 5.1.1. Bifactorial ANOVA Analysis for MTT Assay and Caspase-3 Activity

##### MTT

With regard to the MTT assay for cell viability analysis, once the homogeneity of variance was confirmed with the normality test (*p* = 0.21, n.s.), we performed a bifactorial ANOVA analysis using SigmaPlot 11.v11, confirmed not only a significant effect of LPS -inflammation factor- [LPS: F (1,8) = 669.03, *p* = 0.009], but also a significant effect for fractalkine treatment alone -neuroprotection factor- [sFK: F (1,8) = 12, *p* < 0.01], including a significative interaction between both factors [LPS * sFK, F (1,8) = 205, *p* < 0.001].

For the MTT assay, the Bonferroni post hoc confirmed increased apoptosis by the LPS factor associated with caspase-3 overactivation compared with untreated controls (*p* = 0.003). Conversely, sFK treatment decreased caspase-3 activity in LPS-treated neurons compared with LPS treatment alone (*p* < 0.001).

Finally, the power (beta) of the performed test with alpha = 0.05 for each factor is as follows:

Beta with alpha = 0.05 for LPS: 0.83;

Beta with alpha = 0.05 for FK: 1;

Beta with alpha = 0.05 for LPSxFK: 1.

##### Caspase3 Activity

Once the equal variance test was passed (*p* = 0.216, n,.s) for caspase-3 activation, we performed a bifactorial ANOVA analysis and Bonferroni post hoc for multiple comparisons. The bifactorial ANOVA indicated a significant effect for LPS factor -inflammation- [LPS: F (1,8) = 169, *p* < 0.001], a significant effect of fractalkine factor -neuroprotection- [sFK: F (1,8) = 74.5, *p* < 0.01), as well a significant interactive effect for both factors [LPS * sFK: F (1,8) = 25, *p* = 0.001]. In addition, the power (beta) of the performed test with alpha = 0.05 for each factor is as follows:

Beta with alpha = 0.05 for LPS: 1;

Beta with alpha = 0.05 for FK: 1;

Beta with alpha = 0.05 for LPSxFK: 0.99.

The Bonferroni-corrected analysis confirmed higher caspase-3 activity in LPS-treated cells than untreated controls (*p* = 0.003), while sFK recombinant treatment decreased caspase-3 activation in LPS-treated neurons as compared to LPS treatment alone (*p* < 0.001).

## 6. Discussion

Our study aimed to investigate whether certain peripheral biomarkers could distinguish and/or differentiate between AD and FTD cognitive impairments. NfL M is considered a biomarker of neuroaxonal injury, and its levels in plasma and CSF increase with age, and even more so in neurodegenerative disorders such as AD and FTD, often before cognitive symptoms appear [5]. However, NfL M’s sensitivity to neuroaxonal damage limits its usefulness in differentiating dementia subtypes due to its limited disease specificity [33]. In our study, plasma NfL M levels did not differ between AD and FTD patients, which is consistent with its limited etiological specificity [5]. Since high peripheral NfL M levels are characteristic of aging [33], this biomarker is considered more informative in individuals younger than 60 years, where it can better support or exclude a neurodegenerative diagnosis [33]. A recent study indicates that elevated plasma levels of NfL M and GFAP were both associated with increased mortality risk in FTD [34]. However, in cognitively healthy individuals, higher plasma NfL M levels are associated with age-related comorbidities [35], which may obscure group differences in older cohorts [36].

We went further and quantified p-Tau217 in individuals with AD, FTD, and HIV-1 seropositivity (without cognitive impairment) as a potential biomarker for cognitive impairment. Previous clinical studies in AD have published data on this topic [37]. The elevated plasma p-Tau217 levels found in patients with AD and FTD compared with age-matched, cognitively healthy controls are consistent with previous reports [37,38,39,40,41,42]. These p-Tau217 elevations could predict amyloid pathology in AD patients with accuracy similar to CSF levels [38,42,43]. Several studies have reported an association between p-Tau217 and amyloid positron emission tomography [43]. P-tau assays have demonstrated strong potential as biomarkers in clinical trials and observational studies [40,41,42,43,44,45,46]. In fact, CSF p-Tau217 levels can distinguish between AD and non-AD disorders with 91% accuracy [41]. In this way, Varela et al. reported elevated p-Tau217 levels in a patient cohort [45]. However, their study included fewer FTD patients than our cohort. Thus, peripheral p-Tau217 levels may be associated with cognitive impairment in FTD and AD in our study, which is consistent with the peripheral p-tau217 elevations reported in women over 70 years old who are carriers of *APOE* ε4 (a genetic risk factor for AD) [46].

On the other hand, it is known that p-tau217 accumulates in the hippocampus of a rodent tauopathy model, which supports its relevance in both human and animal studies [47]. Thus, peripheral p-Tau217 levels may serve as a biomarker for cognitive impairment, including AD or FTD patients, in our cohort. The TRAILBLAZER-ALZ (NCT03367403) clinical trial suggests that plasma p-Tau217 levels can be used to monitor neurofibrillary tangle burden and amyloid plaque accumulation in patients [47,48,49,50]. However, in our study, p-tau217 levels did not differ between HIV-1 seropositive individuals (without cognitive impairment) and control subjects. Collectively, these elevated peripheral p-tau217 levels in AD are consistent with its proposed role as a peripheral biomarker of cognitive impairment and brain atrophy in AD [51,52,53,54,55,56,57,58,59,60]. These augmented p-tau217 levels in AD or FTD patients are consistent with the notion that p-tau217 accumulation could reflect their cognitive impairment [51,52]. In this way, elevated plasma p-Tau217 levels in FTD are linked to brain atrophy in AD [54,55,56,57,58,59,60]. Furthermore, the cellular localization of p-Tau217 in the brain has been reported to be associated with plasma p-tau217 levels [18]. In addition, p-tau217 reflects tau tangle pathology [55], and p-tau217 deposition may help distinguish AD pathology from primary age-related tauopathy and other non-AD tauopathies [56,57,58,59,60]. In this regard, p-tau217 is undetectable in normal brain tissue but is detected in the brains of AD patients surrounding areas with amyloid-β plaques [17]. However, p-tau217 levels do not differ between HIV-1 and age-matched controls, a condition associated with altered immune and adaptive responses [23]. However, our findings do not exclude its potential role as a peripheral biomarker associated with cognitive impairment compared with control subjects (without neurodegeneration).

Among Aβ-positive individuals, p-tau217 levels predicted cognitive impairment more accurately than p-tau181 levels [61,62,63,64]. Thus, the presence of tau deposits was observed in the hippocampus of post-mortem FTD brains together with hippocampal involution and other signs of neurodegeneration. In fact, our histopathological analysis revealed pronounced microglial activation and p-tau217 accumulation, supporting the involvement of the hippocampus and broader regional alterations, including cortical and entorhinal areas (see Figure 3). In one study, p-Tau217 was also reported to correlate with lower cognitive performance before the onset of explicit symptoms of the disease [63].

Instead, it is known that CX3CR1/fractalkine chemokines exert a dual protective role but also contribute to cognitive impairment in AD [64,65]. The sFK, also known as CX3CL1, is a released chemokine from the membrane of vulnerable neurons; sFK can signal through the CX3CR1 receptor on microglia and peripheral leukocytes, and this CX3CR1/sFK axis plays an important role in neuroimmune communication [64].

As a novelty, we present the first evidence here that elevated peripheral sFK levels may also predict cognitive dysfunction in FTD. Previous studies have reported that both sFK plasma levels and p-tau217 are clinical biomarkers of cognitive impairment in AD [63] and other neurological conditions [54,55,64]. Under pathological conditions associated with inflammation, sFK is cleaved from the neuronal form and released as a soluble form [21,65]. Depending on the cellular context, sFK can be neuroprotective or neurotoxic [42,65]. Notably, the significant correlation between CX3CR1 and its soluble fractalkine ligand suggests a coordinated systemic immune response linked to cognitive function in patients with FTD in our study. However, fractalkine promotes neuroprotection against LPS-induced inflammation in cortical neurons at 7 DIV in our study. It has been proposed that FK contributes to the initial cognitive decline in AD by promoting central nervous system (CNS) injury and neuroinflammatory cascades [63,64].

This could support the idea that the soluble fractalkine isoform plays a detrimental role in our cohort of FTD patients. We should consider whether fractalkine exerts a dual neuroprotective or neurodegenerative role, depending on the cellular context in cases of inflammation and metalloprotease over-activation [21]. Conversely, other evidence supports the neuroprotective role associated with fractalkine signaling through the CX3CR1 chemokine receptor [62]. In our study, recombinant sFK treatment attenuated caspase-3 activation in LPS-treated cortical neurons at 7 DIV, supporting its neuroprotective role under inflammatory stress [42,65]. Systemic increases in sFK in FTD are consistent with the elevated plasma levels reported in AD [63]. However, peripheral CX3CR1/sFK levels did not distinguish between AD and FTD in our study. The CX3CL1/CX3CR1 axis exhibits context-dependent effects because CX3CR1 deletion mitigates neuronal loss in AD transgenic mice [66] and reduces Aβ deposition [67]. In our cohort, CX3CR1 and sFK levels did not differ between HIV-1 seropositive individuals and healthy subjects without neurodegeneration. This suggests that delta chemokine elevations are more closely related to cognitive dysfunction in FTD. Conversely, sFK elevations may reflect a compensatory response to inflammatory stress, as occurs in LPS-exposed cortical neurons. In this context, peripheral monocytes can amplify neuroinflammatory responses once they reach the brain parenchyma [67]. We cannot rule out the possibility that metalloprotease-mediated sFK release can exacerbate inflammation through the infiltration of monocytes into the brain of FTD patients. Consistent with the protective role of this pathway (CX3CR1/sFK axis), our study found that CX3CR1 blockade increased caspase-3 activation in cortical neurons. In fact, IL-1β blockade and loss of CX3CR1 signaling enhance tau hyperphosphorylation and aggregation, accompanied by increased sFK release as a potentially compensatory response [68]. Because endothelial cells produce sFK and CX3CR1 is expressed on circulating monocytes/leukocytes, even in healthy subjects [69], the elevated CX3CR1/sFK levels found in plasma remain a plausible therapeutic target for modulating neuroinflammation and, consequently, Aβ and tau pathologies. In fact, imbalances in sFK signaling via CX3CR1 are more closely linked to astrogliosis, microglial overactivation, and/or neuronal dysfunction in mild cognitive impairment [70]. However, in our study, we could not determine whether these increased levels contribute to cognitive impairment in FTD or reflect compensatory protective responses against brain damage.

Additionally, sFK contributes to the trafficking of HIV-infected lymphocytes into the brain in HIV-associated neurocognitive disorders [71]. Since sFK does not contribute to cognitive dysfunction in seropositive patients with suppressed viral loads, we cannot exclude the possibility that increased CX3CR1/sFK signaling influences tau accumulation in the hippocampus in FTD, given that sFK overexpression suppresses tau pathology in a murine model of tauopathy [72]. In our study, patients with both AD and FTD exhibited high sFK levels, which could facilitate their stratification for therapeutic interventions with neuroprotectants in clinical trials targeting AD or FTD pathology. The release of soluble fractalkine by damaged neurons under inflammatory conditions limits microglial neurotoxicity. In fact, metalloproteases, such as ADAM10 and ADAM17, have been demonstrated to cleave membrane fractalkine into its soluble form under inflammatory conditions [64]. However, sustained fractalkine release promotes neuroprotection and astroglial/microglial surveillance, yet it also contributes to neurodegeneration [62]. This indirect evidence is consistent with the anti-apoptotic role of sFK against LPS-induced inflammation found in cortical neurons at 7 DV in our study. Indeed, fractalkine protects hippocampal neurons against LPS-induced neurotoxicity, and this effect is blocked by anti-CX3CR1 antibodies in our in vitro study. Therefore, fractalkine counteracts caspase-3 overactivation in LPS-treated neurons and increases neuronal survival. Our findings are consistent with the suppression of tau pathology in a murine model of tauopathy by fractalkine overexpression [72]. Further studies will examine whether metalloprotease activation through fractalkine release promotes neuroprotection or contributes to cognitive impairment in FTD.

However, the relationship between chemokine-dependent inflammation and TDP-43 translocation from the nucleus to the cytoplasm is unknown in FTD patients. TDP-43 is a DNA/RNA-binding protein necessary for RNA metabolism. It forms cytoplasmic aggregates in neurons and glial cells and accumulates in the cytosol in the hippocampus under neuropathological conditions. TDP-43 appears to be a more specific marker for FTD patients [73,74], whereas they are typically unaffected in AD [75]. TDP-43 peripheral levels did not differ between AD patients and cognitively healthy controls. Although no positron emission tomography (PET) tracers for cerebral TDP-43 exist, increased brain accumulation of TDP-43 is consistently associated with poorer cognition. In fact, TDP-43 can distinguish FTD cases with ubiquitin/TDP-43 pathology from those with tauopathy [76]. Consistent with our blood findings, plasma levels of total and/or phosphorylated TDP-43 in FTD pathology correlated with brain TDP-43 [77,78].

## 7. Conclusions

The ability to interpret short-term changes in patients with mild cognitive impairment is crucial for identifying disease-modifying therapies for FTD and AD. Thus, our findings underscore the promising utility of peripheral CX3CR1/sFK levels as protein signatures to estimate cognitive impairment in AD or FTD, thereby facilitating screening and intervention strategies. Since AD begins 20 years or more before symptoms appear, there is a substantial window of opportunity to identify peripheral biomarkers of cognitive impairment without an invasive technique. For the first time, we have identified a role for soluble fractalkine as a potential FTD biomarker of cognitive impairment; however, these high levels could reflect compensatory responses against neuronal damage in FTD patients. Taken together, high levels of these plasma chemokines (p-Tau217 and CX3CR1/sFK) may serve as valid biomarkers of cognitive dysfunction, thereby preventing unnecessary lumbar punctures. Our novel findings suggest that increased sFK levels may be associated with FTD pathology. In addition, the proposed role of TDP-43 as a biomarker of FTD pathology is consistent with previous evidence showing that TDP-43 expression exacerbates FTD-related phenotypes in progranulin-insufficient mice [79]. Overall, these biomarkers could identify patients at risk for dementia who require PET scans or CSF aspiration to assess concomitant dementia biomarkers.

## 8. Limitations and Future Perspectives

Although plasma biomarkers have been included in recent recommendations from the Global CEO Initiative on Alzheimer’s Disease, their implementation in clinical practice is necessary to demonstrate their usefulness [80]. Future studies should evaluate how plasma p-tau levels and other dementia biomarkers vary by age, sex, body mass index (BMI) [81,82], or comorbidities [83]. In addition, the influence of comorbidities (hypertension, obesity, and type 2 diabetes, etc.) on inflammatory mediators related to aging and infections in patients with AD or FTD will be evaluated in the future. Finally, standardization of biofluid assays, tau PET quantification methods, and cut points are necessary in clinical studies [84]. Another important issue is addressing the influence of additional pathologies, such as chronic renal disease, which increases peripheral p-tau217 levels [85,86].

In general, given the well-known age dependence of several plasma biomarkers, particularly NfL M, this may introduce bias in clinical studies. Further studies from our group will study whether metalloprotease activation plays a neuroprotection/neurotoxic role through fractalkine release. The co-existence of cerebrovascular disease and Lewy body disease with AD pathology will also be evaluated by our group in the future.

## Figures and Tables

**Table 1:** Demographic and cognitive evaluation by Mini-Mental scores of patients (* *p* < 0.05).

Variables	Age-Matched Controls	AD	FTD	*p*	
**Size samples** **Number of women**	53	53	53	-	
	21 ± 9	21 ± 7	34 ± 8	-	
**BMI index** **(kg/m^2^)**	22.5 ± 4	21.34 ± 6	23 ± 8	*p* = 0.4	
**Education** **(years)**	7 ± 2	6 ± 3	6.4 ± 1	*p* = 0.38	
**Age of onset** **(years)**		68 ± 7	70 ± 6.8	*p* = 0.9	
**mini mental scores** **(MMSE test)**	29 ± 2.1	15 ± 0.52 *	14.1 ± 0.46 *	* *p* < 0.05	AD or FTD vs. cont

This observational study (without clinical interventions) enrolled 227 patients recruited by a general neurologist. The AD patients met the NINCDS-ADRDA criteria for probable AD and had MMSE (Mini-Mental State Examination) scores) lower than controls (*p* < 0.05 vs. control), and FTD patients had lower scores than controls (*p* < 0.05). BMI and educational level did not differ between study groups with cognitive impairment (AD or FTD) and the age-matched control groups (*p* > 0.05, n.s.). Their cognitive impairments were confirmed by their significantly lower scores on the MMSE (adapted to the Spanish scale) than the control group (without cognitive impairment, *p* < 0.05 vs. control). Sociodemographic variables considered included age, sex, and education level (classifying subjects with primary, secondary, or higher education). These variables were not significant for the cognitive status of AD or FTD patients (*p* > 0.05, n.s.).

**Table 3:** Foc ROC curves (n.s. not significant).

Range of Detection by ELISA		ROC Curve AUC Values and 95% of CI
CX3CR1 (15.5 pg/mL)	AD vs. cont	AUC = 0.99, *p* < 0.001 (95% of CI: 0.98 to 1)
	FTD vs. cont	AUC = 0.99, *p* < 0.001 (95% of CI: 0.99 to 1)
	FTD vs. AD	AUC = 0.58, *p* < 0.14, n.s. (95% of CI: 0.41 to 0.69)
sFK (78 pg/mL)	AD vs. cont	AUC = 0.61, *p* = 0.046 (95% of CI: 0.51 to 0.72)
	FTD vs. cont	AUC = 0.68, *p* = 0.011 (95% of CI: 0.58 to 0.78)
	FTD vs. AD	AUC = 0.53, *p* = 0.4, n.s. (95% of CI: 0.42 to 0.64)
**TDP-43** (0.0468 ng/mL)	AD vs. cont	AUC = 0.6, *p* < 0.05 (95% of CI: 0.49 to 0.71)
	FTD vs. cont	AUC = 0.7, *p* = 0.004 (95% of CI: 0.6 to 0.79)
	FTD vs. AD	AUC= 0.61, *p* < 0.039 (95% of CI: 0.51 to 0.72)
**p-tau217** (0.32 pg/mL)	AD vs. cont	AUC = 0.96, *p* <0 001 (95% of CI: 0.91 to 1)
	FTD vs. cont	AUC = 0.94, *p* < 0.001 (95% of CI: 0.92 to 1)
	FTD vs. AD	AUC= 0.52, *p* < 0.67, n.s. (95% of CI: 0.41 to 0.63)
**GFAP** (9.38 pg/mL)	AD vs. cont	AUC = 0.99, *p* < 0.001 (95% of CI: 0.97 to 1)
	FTD vs. cont	AUC = 0.94, *p* < 0.001 (CI: 0.93 to 0.98)
	FTD vs. AD	AUC= 0.58, *p* < 0.13, n.s. (95% of CI: 0.47 to 0.69)
**NfL M** (5 ng/mL)	AD vs. cont	AUC = 0.92, *p* < 0.0001 (95% of CI: 0.87 to 0.97)
	FTD vs. cont	AUC = 0.96, *p* < 0.001 (95% of CI: 0.93 to 0.99)
	FTD vs. AD	AUC = 0.57, *p* = 0.2, n.s. (95% of CI: 0.46 to 0.68)

## Data Availability

All data analyzed during this study are included in this published article and its Supplementary Information Files. Further inquiries can be directed to the corresponding author (JJM).

## Supplementary Materials

The following supporting information can be downloaded at: https://www.mdpi.com/article/10.3390/life16091554/s1, Supplementary file: ELISA methods.
