# Supplementary material for: Comparative Analysis of Plasma Biomarkers of Alzheimer’s Disease and Frontotemporal Dementia: The Dual Role of Soluble Fractalkine as a Biomarker of Frontotemporal Dementia and Its Neuroprotective Effects in Cortical Neurons “In Vitro”
Source: Life (Basel). 2026 Sep 16;16(9):1554. doi: 10.3390/life16091554 (PMC13608586; doi:10.3390/life16091554)
Supplement: Supplementary file 1 [file life-16-01554-s001.zip › life-4525329-supplementary.pdf]

Supplementary file

ELISA methods

2.2.2. CX3CR1

This sandwich ELISA kit detects human CX3CR1 protein levels ranging from 15.63 to 1000 pg/mL (Novus Biologicals NBP3-0681). Plasma samples were added to the plate and combined with the primary antibody overnight. The plate was washed three times with a washing buffer. Then, a biotinylated conjugate antibody that recognizes human CX3CR1 and avidin-horseradish peroxidase (HRP) was added, and the plate was incubated in a humidity chamber. After wahsing, the substrate solution was added to the wells. The reaction was stopped by adding 2.5 N sulphuric acid, and the optical density (OD) was measured by spectrophotometry at 450 nm using a microplate reader (Thermo Fisher), calculated by interpolation with the standard curve range and expressed in pg/mL.

2.2.3. TDP-43 levels

The TDP43 ELISA Kit (A314391, Antibodies.com company) is a sandwich enzyme immunoassay that quantifies human TDP43 using a pre-coated TDP-43 antibody on a 96-well microtiter plate. The plate is pre-coated with an specific TDP43 antibody. The standards and plasma samples are added into the wells, where TDP43 binds to the immobilized antibody. The biotinylated anti-TDP43 antibody binds to the TDP43, and streptavidin-HRP fixes to the biotinylated anti-TDP43 antibody, enhancing the signal. After incubation, the unbound biotinylated anti-TDP43 antibody and streptavidin-HRP are removed by washing. Two substrate solutions are added to the wells, and the labelling colour develops in relationship to the amount of TDP43 captured in each well. The reaction was stopped by adding a stop solution that turn the blue colour into yellow. The TDP43 concentration in the samples was calculated by measuring the absorbance at 450nm using a microplate reader and referencing the standard curve. This ELISA method can detect serum, plasma or other biological fluids in samples with a sensitivity of 0.048 ng/mL within a range of 1.5-24 ng/mL.

2.2.4. p-tau217

Plasma p-tau217 levels were measured using a Simple Plex™ immunoassay for the detection of human phospho-tau in plasma (Simple Plex Ultra-Sensitive Human pTau217 ALZpath Assay, R&D Systems, Catalog # SPCKB-PS-015536 Human Phospho-tau T217, ALZpath, EDTA/Heparin, R&D, USA, D10-1346-001/Rev A), with 2-fold dilution of the buffer containing 35 µL to 35 µL of the sample for human phospho-tau (p-Tau217). The standard curve was prepared at 150 ng/mL in diluent buffer and assayed for cross-reactivity. The assay used is specific for phosphorylation at Thr217 using a monoclonal capture antibody specific for p-Tau217 (Thr217) was coated onto the wells of the 96-well plate. During the first incubation, standards of known concentrations and unknown samples were pipetted into the wells, and the antigens bound to the immobilized capture antibody. After washing, a rabbit antibody specific for p-Tau217

served as a detection antibody captured during the first incubation. After washing, a horseradish peroxidase-labelled anti-rabbit IgG was added. After a third incubation and three washes to remove the unbound enzyme, a substrate solution (TMB) was added, and the optical density (OD) was measured an determined in a standard microplate reader. The quantitation limit was 0.32 pg/mL.

2.2.5. Neurofilament M (NfL M)

The Human Neurofilament Heavy Polypeptide ELISA Kit (A310593; antibodies.com) uses a sandwich system to quantitatively measure human NfL M in serum, plasma, and other biological fluids. An antibody specific for Nfl was pre-coated onto a 96-well microtiter plate. The standards and test samples are added to the wells, where NfL M binds to the immobilized antibody. Then, biotinylated anti-Nfl binds to the NfL M in each well, and streptavidin-HRP binds to the biotinylated anti-NfL M. After an incubation period, any unbound biotinylated NfL M and unbound streptavidin-HRP are removed by washing. Then, two substrate solutions are added to the wells. The color that develops is proportional to the amount of NfL M determined. Finally, the stop buffer changes the color from blue to yellow. The NfL M concentration is calculated by reading OD at 450nm on a microplate reader and interposing it within the standard curve. The limit of detection is between 5-80 ng/mL.

2.2.6. Glial Fibrillary Acidic Protein (GFAP)

The sensitivity for GFAP detection is 9.38 pg/mL, and the detection range is between 15.63-1000 pg/mL for human samples using the ELISA Sandwich method (E-EL-H6093, ELabscience, Human GFAP Kit). Standards and plasma samples are added to the plate wells and combined with the specific antibody. Briefly, the plate is pre-coated with an antibody specific to human GFAP. Then, a biotinylated detection antibody recognizes GFAP, and avidin-horseradish peroxidase (HRP) conjugate enhances the signal. After five washing steps, the colorimetric substrate solution is added to each well. The reaction is stopped by adding the stop solution. The OD was measured at 450 nm using a spectophotomer (Thermo), and the concentration is calculated by interposing within the standard curve.
